# Identification of proteins and cellular pathways targeted by 2-nitroimidazole hypoxic cytotoxins

**DOI:** 10.1016/j.redox.2021.101905

**Published:** 2021-02-21

**Authors:** Faisal Bin Rashed, Alexandru Cezar Stoica, Dawn MacDonald, Hassan El-Saidi, Carolynne Ricardo, Bhumi Bhatt, Jack Moore, Diana Diaz-Dussan, Nirilanto Ramamonjisoa, Yvonne Mowery, Sambasivarao Damaraju, Richard Fahlman, Piyush Kumar, Michael Weinfeld

**Affiliations:** a Department of Oncology, University of Alberta, Edmonton, AB, T6G2R3, Canada; b Department of Pharmaceutical Chemistry, Faculty of Pharmacy, University of Alexandria, El Sultan Hussein St. Azarita, Alexandria, Egypt; c Department of Laboratory Medicine & Pathology, University of Alberta, Edmonton, AB, T6G2R3, Canada; d Alberta Proteomics and Mass Spectrometry Facility, University of Alberta, Edmonton, AB, T6G2R3, Canada; e Department of Chemical & Materials Engineering, University of Alberta, Edmonton, AB, T6G2R3, Canada; f Radiation Oncology, School of Medicine, Duke University, Durham, NC, 27708, United States; g Department of Biochemistry, University of Alberta, Edmonton, AB, T6G2R3, Canada

**Keywords:** Hypoxia, Nitroimidazole, Click chemistry, Head and neck tumour, Proteomics

## Abstract

Tumour hypoxia negatively impacts therapy outcomes and continues to be a major unsolved clinical problem. Nitroimidazoles are hypoxia selective compounds that become entrapped in hypoxic cells by forming drug-protein adducts. They are widely used as hypoxia diagnostics and have also shown promise as hypoxia-directed therapeutics. However, little is known about the protein targets of nitroimidazoles and the resulting effects of their modification on cancer cells. Here, we report the synthesis and applications of azidoazomycin arabinofuranoside (N_3_-AZA), a novel click-chemistry compatible 2-nitroimidazole, designed to facilitate (a) the LC-MS/MS-based proteomic analysis of 2-nitroimidazole targeted proteins in FaDu head and neck cancer cells, and (b) rapid and efficient labelling of hypoxic cells and tissues. Bioinformatic analysis revealed that many of the 62 target proteins we identified participate in key canonical pathways including glycolysis and HIF1A signaling that play critical roles in the cellular response to hypoxia. Critical cellular proteins such as the glycolytic enzyme glyceraldehyde-3-phosphate dehydrogenase (GAPDH) and the detoxification enzyme glutathione S-transferase P (GSTP1) appeared as top hits, and N_3_-AZA adduct formation significantly reduced their enzymatic activities only under hypoxia. Therefore, GAPDH, GSTP1 and other proteins reported here may represent candidate targets to further enhance the potential for nitroimidazole-based cancer therapeutics.

## Highlights

• Azidoazomycin arabinofuranoside (N_3_-AZA) is a novel hypoxia-targeting compound designed to undergo click chemistry.
• We report 62 protein targets of N_3_-AZA; many of which are critical for cellular response to hypoxia, such as GAPDH and GST.
• Binding of N_3_-AZA reduced GAPDH and GST enzyme activity, indicating that nitroimidazoles can disrupt key cellular pathways.
• Fluorescent imaging with N_3_-AZA click chemistry is highly efficient for mapping cellular and tissue hypoxia.
• No relationship was observed between tumour size and hypoxia.

## 1 Introduction

Rapid growth of cancer cells, coupled with chaotic tumour vasculature, creates regions within the tumour where cells are deprived of nutrients and oxygen [1]. This phenomenon, clinically termed “tumour hypoxia”, has been reported in almost all solid malignancies. Cells residing in these hypoxic niches up-regulate a myriad of pro-survival adaptive responses including impaired drug delivery [2], oncogene activation [3], abnormal cellular metabolism [4] and deregulated DNA damage checkpoint signalling [5]. Furthermore, low O_2_ tension stimulates metastasis, invasion and relapse [6], and sustains growth and maintenance of tumour initiating cells [7]. Most importantly, these adaptations make hypoxic cancer cells resistant to radiation, chemotherapy and immunotherapy, resulting in poor patient prognosis [8,9]. While this rationalizes the need for hypoxia directed therapy, it has yet to become a part of routine clinical practice.

Nitroimidazoles (NIs) are a class of hypoxia activated prodrugs that are bioreductively activated in an inverse O_2_-dependent manner. The process involves reduction by nitroreductases that generates nitroradical anions; this intermediate is re-oxidized in the presence of O_2_, allowing the drug to freely diffuse out of well-oxygenated cells. Under hypoxia, the nitro-radical anion undergoes reductive decay producing a reactive hydroxylamine, which covalently binds to cellular macromolecules, leading to hypoxia-selective accumulation of the drug [10]. This unique property makes NIs ideal for targeting hypoxic tumour cells, both as cytotoxins and radiosensitizers. However, despite showing early promise, several NIs failed in clinical trials as hypoxia directed cancer therapeutics [11,12]. These disappointing outcomes can be attributed, in part, to our limited understanding of the precise molecular mechanism of NIs. Indeed, the protein targets of NIs in a human cancer context have never been explored, making it difficult to comprehend their effects on target proteins. Here, we outline a strategy to label and isolate NI target proteins using a novel click chemistry compatible 2-NI agent, azidoazomycin arabinofuranoside (N_3_-AZA). The proteins were then characterized using liquid chromatography coupled mass spectrometry (LC-MS/MS). Bioinformatics analysis revealed that many of these proteins play critical roles in the cellular response to hypoxia.

The modular nature of N_3_-AZA click reaction also supported its use as an excellent hypoxia marker. 2-NIs are widely used as hypoxia diagnostics, both in radiologic imaging and in immune-based detection. Positron emission tomography (PET) scans using radiolabelled 2-NIs, such as ^18^F-fluoromisonidazole (^18^F-FMISO) or ^18^F-fluoroazomycin arabinoside (^18^F-FAZA), allow for non-invasive functional imaging of tumour hypoxia [13,14]. The current histological test for examining and evaluating tumour hypoxia involves the administration of 2-NI compounds, such as pimonidazole (Hypoxyprobe™) and EF5 [2-(2-nitro-1*H*-imidazol-1-yl)-*N*-(2,2,3,3,3-pentafluoropropyl) acetamide], and immunochemical detection of drug-protein adducts on tissue biopsies [15,16]. In contrast, N_3_-AZA click chemistry offers an antibody-independent alternative by using fluorescently tagged alkynes to visualize histological hypoxia. The result is a hypoxia-selective histochemical reagent that is simple, precise and time efficient.

## 2 Materials and methods

**General synthesis methods:** Solvents used in reactions were purified before use by successive passage through columns of alumina and copper under an argon atmosphere. All reagents were purchased from commercial sources and were used without further purification unless noted otherwise. All reactions were carried out under a positive pressure argon atmosphere and monitored by thin-layer chromatography (TLC) on Silica Gel G-25 UV254 (0.25 mm) unless stated otherwise. TLC spots were detected under UV light and/or by charring with a solution of anisaldehyde in EtOH, acetic acid and H_2_SO_4_. Column chromatography was performed on Silica Gel 60 (40–60 mm). Organic solutions were concentrated under vacuum at <50 °C. ^1^H and ^13^C NMR spectra were recorded at 400 and 101 MHz, respectively. ^1^H and ^13^C NMR chemical shifts are referenced to CD_3_OD (d = 3.35 and 4.78 for 1H, 48.9 for 13C). ^1^H NMR data are reported as though they are first order and the peak assignments were made on the basis of 2D-NMR (^1^H–^1^H COSY and HMQC) experiments. ESI-MS spectra were obtained on samples suspended in CH_3_OH with added NaCl.

Synthesis of N_3_-AZA was explored from two precursors, namely 1-α-D-(5-*O*-tosyl-arabinofuranosyl)-2-nitroimidazole (Ts-AZA) [17] [See supporting materials] and 1-α-D-(5-deoxy-5-iodo-arabinofuranosyl)-2-nitroimidazole (IAZA) [18]. The IAZA route described here showed better yield and accommodated the increase in the reaction temperature to 100 °C without producing detectable side products. Briefly, NaN_3_ (0.33 g, 10.2 mmol) was added to a solution of IAZA (1.8 g, 5.1 mmol) in DMF (15 mL) and the reaction mixture was heated at 100 °C for 2 h. After cooling to room temperature, the reaction mixture was concentrated under vacuum and quenched with H_2_O (20 mL). The product was extracted into EtOAc (3 × 10 mL) and the organic layer was dried over anhydrous Na_2_SO_4_, filtered and the solvent was removed under reduced pressure. The crude product was purified using a short column chromatography (10:1, *v/v*, CH_2_Cl_2_/CH_3_OH) to give N_3_-AZA (1.4 g, 93%) as an amorphous pale yellow solid: *R*_*f*_, 0.39 (10:1 CH_2_Cl_2_/CH_3_OH); ^1^H NMR (400 MHz, CD_3_OD): δ 7.65 (d, *J* = 1.3 Hz, 1H, H-5Im), 7.12 (d, *J* = 1.3 Hz, 1H, H-4Im), 6.46 (d, *J* = 1.5 Hz, 1H, H-1), 4.54 (ddd, *J* = 7.5, 5.0, 2.5 Hz, 1H, H-2), 4.28 (t, *J* = 1.8 Hz, 1H, H-3), 4.06 (t, *J* = 2.3 Hz, 1H, H-4), 3.63 (dd, *J* = 12.9, 7.4 Hz, 1H, H-5), 3.47 (dd, *J* = 12.9, 5.0 Hz, 1H, H-5); ^13^C NMR (101 MHz, CD_3_OD) δ 140.02 (C-2Im), 126.76 (C-4Im), 123.83 (C-5Im), 95.56 (C-1), 88.48 (C-2), 82.38 (C-3), 77.12 (C-4), 52.29 (C-5); HRMS (ESI) calcd for [M+Na]^+^ C_8_H_10_N_6_O_5_Na: 293.0605, found: 293.0604.

**Cell culture:** Human head and neck squamous cell carcinoma cell line, FaDu, was purchased directly from American Type Culture Collection (ATCC HTB-43, Manassas VA). Additional cell lines used include A549 (human lung epithelial carcinoma), PC3 (human prostate carcinoma) [both purchased directly from ATCC] and A172 (human glioblastoma) [kindly provided by Dr. Roseline Godbout, University of Alberta, Canada]. Cells were expanded and frozen at early passage in liquid nitrogen until used. Cells were grown as monolayer cultures in DMEM/F-12 media supplemented with 10% fetal bovine serum, 1% of 2 mM l-glutamine and 1% penicillin streptomycin. Prior to initiation of experiments, cells were tested for mycoplasma contamination by Hoechst 33342 staining (Life Technologies) and confocal imaging. Cell cultures were maintained in a humidified incubator at 37 °C with 5% CO_2_, for a maximum of 2 months (~25–30 passages). Hypoxic incubations were carried out in a humidified chamber under controlled O_2_ flow (ProOx P110, BioSpherix, Parish, NY). O_2_ levels below 0.1% were achieved using an in-house degassing/regassing system [19].

**Drug stock preparation:** N_3_-AZA and pimonidazole hydrochloride (hereafter referred to as pimonidazole; Hypoxyprobe™, Burlington, MA) were prepared as 500 μM and 656 μM solutions in DMSO respectively and stored at -20 °C.

**Crystal violet staining assay:** Cells seeded in 96-well plates (Cellstar®; 655180, Sigma-Aldrich, Oakville, ON) at densities ranging from 1500 to 5000 cells per well (depending on the cell line) were left overnight to attach and treated with increasing concentrations of N_3_-AZA or pimonidazole (0 μM to 2000 μM). Drug treatment lasted for 72 h under normoxia (20% O_2_) or hypoxia (0.1% O_2_); 0.02% DMSO was used as vehicle control. Afterwards, media was discarded, attached cells were stained with 0.05% crystal violet (Sigma-Aldrich, C6158), washed and left to dry for 24 h. Crystal violet stained cells were resuspended in 150 μL of methanol and the optical density (OD) was measured at 584 nm using a FLUOstar OPTIMA microplate reader (BMG Labtech, Ortenberg, Germany). Percent cell survival was calculated by subtracting OD of blank wells and then normalizing DMSO controls to 100%. The first point on each curve represents 0 μM drug.

**Click chemistry reaction cocktail preparation:** The Click-IT reaction cocktail (Molecular Probes, Eugene, OR) was made up according to the manufacturer's directions using a 1:500 dilution of 10 mg/mL biotin-alkyne (Cat. #C37B0, Lumiprobe, Hallandale Beach, FL) for proteomic studies or with a 1:5000 dilution of the 2 mg/mL Alexafluor 488-, 555- or 594-conjugated alkyne stock (A10267, A20013, A10275, Molecular Probes) for imaging. Reaction cocktail was used within 10 min of preparation. All reactions were carried out at room temperature in the dark.

**Isolation of N**_**3**_**-AZA-protein adducts:** FaDu cells treated with 100 μM N_3_-AZA (or vehicle control, 0.02% DMSO) for 24 h under normoxia or hypoxia (<0.1% O_2_) were harvested directly in RIPA buffer supplemented with protease inhibitor. Click chemistry was performed on cell lysates at room temperature for 30 min using a biotin-labelled alkyne. Streptavidin-mutein beads were resuspended and processed as per the manufacturer's guidelines (Cat. # Roche 03708152001, Sigma-Aldrich). Clicked lysates (without equilibration buffer) were loaded on 1% BSA blocked streptavidin-mutein beads and put on a rocker overnight at 4 °C. Afterwards, the supernatant was removed, the beads were washed several times, and the bound biotinylated protein fraction was either eluted from the beads using free biotin (Sigma-Aldrich) or by boiling the beads in SDS-PAGE loading dye.

**LC-MS/MS:** Clicked crude extracts and eluates were run on SDS-PAGE, stained with Coomassie G-250 and processed for in-gel trypsin digestion. Briefly, excised gel bands were de-stained twice in 100 mM ammonium bicarbonate/acetonitrile (50:50 v/v). The samples were then reduced in 10 mM 2-mercaptoethanol in 100 mM sodium bicarbonate and alkylated by treatment with 55 mM iodoacetamide in 100 mM bicarbonate. After dehydration, enough trypsin (6 ng/μL, Promega sequencing grade, Madison, WI) was added to just cover the gel pieces and the digestion proceeded overnight (~16 h) at room temperature. Tryptic peptides were first extracted from the gel using 97% water/2% acetonitrile/1% formic acid, followed by a second extraction using 50% of the first extraction buffer with 50% acetonitrile. Fractions containing tryptic peptides were resolved and ionized using a nanoflow HPLC (Easy-nLC II, Thermo Scientific, Waltham, MA) coupled to an LTQ XL-Orbitrap hybrid mass spectrometer (Thermo Scientific). Nanoflow chromatography and electrospray ionization were accomplished using a PicμoFrit fused silica capillary column (ProteoPepII, C18) with 100 μm inner diameter (300 Å, 5 μm, New Objective, Woburn, MA). Peptide mixtures were injected onto the column at a flow rate of 3000 nL/min and resolved at 500 nL/min using a 60 min linear gradient from 0 to 45% v/v aqueous acetonitrile in 0.2% v/v formic acid. The mass spectrometer was operated in data-dependent acquisition mode, recording high-accuracy and high-resolution survey Orbitrap spectra using external mass calibration, with a resolution of 30,000 and *m*/*z* range of 400–2000. The fourteen most intense multiply charged ions were sequentially fragmented by using collision induced dissociation, and spectra of their fragments were recorded in the linear ion trap; after two fragmentations, all precursors selected for dissociation were dynamically excluded for 60 s. Data was processed using Proteome Discoverer 1.4 (Thermo Scientific) and a human proteome database (UniProt) was searched using SEQUEST (Thermo Scientific). Search parameters included a precursor mass tolerance of 10 ppm and a fragment mass tolerance of 0.8 Da. Peptides were searched with carbamidomethyl cysteine as a static modification, and oxidized methionine and deamidated glutamine and asparagine as dynamic modifications. Protein IDs were manually analyzed, and unreviewed UniProtKB/TrEMBL IDs were substituted with respective reviewed UniProtKB/Swiss-Prot IDs for downstream data analysis.

**Bioinformatics analysis:** Proteins identified through LC-MS/MS were plotted as a Venn diagram using the FunRich network analysis tool [20]. An enrichment profile was generated by comparing the relative ion intensities of individual proteins in lysates and eluates from N_3_-AZA treated hypoxic cells, and plotted using GraphPad Prism V7. Canonical protein sequences of putative N_3_-AZA target proteins were retrieved from the UniProt database and analyzed for cysteine (Cys) and proline (Pro) content. Functional categorization of target proteins was performed using gene ontology by PANTHER classification system (biological processes, molecular activity, and cellular components) [21]. The proteomic data set was submitted into Ingenuity Pathway Analysis (IPA) for core analysis [22]. IPA calculates a distinct p-value (p-value of overlap calculated using Fisher's Exact Test) which depicts the association between the protein molecules in our dataset to the literature-reported molecules in IPA knowledgebase. The identified canonical pathways were ranked based on P value. Upstream regulatory analysis was performed to identify the N_3_-AZA target proteins regulated by common regulators.

**Western blot:** N_3_-AZA treated FaDu cells (0 μM, 100 μM and 250 μM, 24 h) were harvested in RIPA buffer supplemented with protease inhibitor. Crude protein extracts were separated on a 10% polyacrylamide gel for 55–70 min at 180 V and transferred onto nitrocellulose membranes. Total protein was visualized using Revert™ 700 Total Protein Stain (926–11016, LI-COR, Lincoln, NE). Membranes were blocked for 1 h with 5% milk [in phosphate-buffered saline (PBS), 0.1% Tween 20] and probed with the following primary antibodies: anti-hypoxia inducible factor 1 alpha (1:2000 dilution; NB100-449, Novus Biologicals), anti-beta tubulin (1:4000 dilution; ab6046, Abcam), anti-glyceraldehyde-3-phosphate dehydrogenase (1:1000 dilution, ab9482, Abcam), anti-glutathione S-transferase P (1:2000 dilution, ABS1650, Millipore), anti-beta actin (1:2000 dilution; sc1616/sc47778, Santa Cruz Biotechnology) and anti-heat shock protein 90 (1:2000 dilution; ab13492, Abcam). Secondary antibodies used include goat anti-rabbit-HRP (1:2000 dilution; Jackson Immunoresearch, West Grove, PA), IR 800 goat conjugated anti-mouse (1:2000 dilution, 926–80010, LI-COR) and IR 800 conjugated goat anti-rabbit (1:2000 dilution; 926–32211, LI-COR). For clicked lysates and eluates, membranes were probed with Streptavidin-HRP (1:2000 dilution, RPN1231V; GE Healthcare Life Sciences, Mississauga, ON). Membranes were then processed with West Pico PLUS chemiluminescent substrate (Thermo Fisher Scientific, Edmonton, AB) and developed on film, or scanned with an Odyssey Fc imager (LI-COR).

**Glyceraldehyde-3-phosphate dehydrogenase (GAPDH) activity assay:** The activity of cellular GAPDH enzyme was quantified using a GAPDH activity assay kit (Abcam, ab204732). Briefly, FaDu cells grown on 60-mm glass petri dishes were treated with either N_3_-AZA (100 μM or 250 μM) or vehicle control (0.02% DMSO) for 24 h under normoxia or hypoxia (<0.1% O_2_). Afterwards, attached cells were harvested by trypsinization, counted, pelleted by centrifugation and dissolved in GAPDH assay buffer (1 × 10^6^ cells per 0.5 mL buffer); 25 μL cell lysate was used to measure GAPDH enzymatic activity using 96 well plates according to the manufacturer's protocol. Absorbance was measured at 450 nm in kinetic mode with a FLUOstar OPTIMA microplate reader; a total of 30 readings were recorded over 48.3 min. GAPDH activity was calculated according to the manufacturer's guidelines.

**Glutathione S-transferase (GST) assay:** A GST assay kit (Sigma, CS0410) was used to measure the enzymatic activity of total cellular GST. In short, FaDu cells treated similarly as described above (GAPDH assay) were scraped, resuspended in 10 ml PBS, counted, and pelleted by centrifugation. The cell pellet was dissolved in PBS containing 2 mM EDTA (1 × 10^6^ cells per 0.1 mL PBS-EDTA) and sonicated. After centrifugation, 10 μL of supernatant was used to measure GST activity in a 96-well plate using the manufacturer's protocol. Absorbance was measured at 340 nm in kinetic mode using a FLUOstar OPTIMA microplate reader; a total of 15 readings were recorded over 20.3 min. GST activity was calculated according to the manufacturer's guidelines.

**N**_**3**_**-AZA click staining of fixed cells**: Cells grown in 35-mm tissue culture plates containing sterilized 22 × 22 cm glass coverslips were incubated with vehicle control (0.02% DMSO) or different concentrations of N_3_-AZA (1 μM, 10 μM or 100 μM) under normoxia (20% O_2_) and hypoxia (1% O_2_ or <0.1% O_2_) for 6 h. Afterwards, cells were washed with PBS and fixed in 2% paraformaldehyde (PFA). Fixed cells were blocked and permeabilized with 1% bovine serum albumin (BSA) in PBS containing 0.1% Triton X-100 for 20 min, followed by incubation with click cocktail containing Alexafluor 488-/555-/594-conjugated alkyne for 30 min at room temperature. Cell nuclei were stained with Hoechst 33342 (1:10,000 dilution in PBS, Life Technologies) for 5 min, washed and mounted on glass slides (Fluoroshield Mounting Medium, Abcam, Cambridge, UK). Images were obtained with a Plan-Apochromat 40X/1.3 Oil DIC lens on a Zeiss 710 confocal microscope using Zen 2011 software (Carl Zeiss, Jena Germany).

**Immunocytochemistry:** Cells treated with N_3_-AZA or pimonidazole or both (at indicated concentrations and duration) under normoxia and hypoxia (<0.1% O_2_) were fixed with PFA and processed for immunocytochemistry. Pimonidazole treated cells (alone or in combination with N_3_-AZA) were stained with a rabbit anti-pimonidazole antibody (1:100 dilution for 2 h; Pab2627, Hypoxyprobe™) and an Alexa Fluor 647 labelled goat-anti-rabbit secondary antibody (1:1000 dilution for 1 h; A21245, Molecular Probes). N_3_-AZA treated cells were processed for the following primary antibodies: anti-nucleolin antibody (1:1000 dilution; ab22758, Abcam or 1:500 dilution; sc17826, Santa Cruz Biotechnology), anti-alpha-tubulin antibody (1:4000 dilution; T6158, Sigma-Aldrich) and anti-glyceraldehyde-3-phosphate dehydrogenase antibody (1:200 dilution; ab9482, Abcam). Alexa Fluor 488 conjugated anti-rabbit (1:1000 dilution; Molecular Probes) and Alexa Fluor 594 conjugated anti-mouse (1:250 dilution; Molecular Probes) secondary antibodies were used for visualizing protein localization. N_3_-AZA treated cells were also processed for actin polymerization using Phalloidin-iFluor 488 reagent (1:1000 dilution; ab176753, Abcam). Click chemistry using an Alexafluor 488-/594-/555-conjugated alkyne (1:5000 dilution; Molecular Probes) was performed only after finishing all antibody staining steps. Cell nuclei were counter-stained with Hoechst (1:10,000 dilution; Life Technologies). Cells stained for actin and tubulin were not processed for click staining. Mounted coverslips on glass slides were imaged as described earlier.

**Tumour generation and drug treatments:** Female BALB/c nude mice (Charles River Laboratories, Wilmington, MA) aged 6 weeks were used and cared for with protocols approved by the Cross Cancer Institute Animal Care Committee (protocol #AC14208) in accord with the Canadian Council on Animal Care guidelines. We subcutaneously injected 6 × 10^6^ FaDu cells in 0.1 mL of sterile 0.9% NaCl into the upper right flank to form palpable tumours. The tumours were measured daily until they reached desired volume, when the mice were divided into 4 groups and injected intraperitoneally (i.p.) with 0.15 mL 0.9% NaCl containing (i) 80 mg/kg N_3_-AZA, (ii) 80 mg/kg pimonidazole, (iii) total 80 mg/kg N_3_-AZA: pimonidazole in a 1:1 ratio and (iv) 0.9% NaCl control. We also used a primary mouse model of HPV-negative squamous cell carcinoma of the oral cavity, developed by Dr. Yvonne Mowery [23]. These animal studies were performed in accordance with protocols approved by the Duke University Institutional Animal Care and Use Committee (IACUC, protocol #A032-19-02) and adhered to the NIH Guide for the Care and Use of Laboratory Animals. In brief, tumours were generated by breeding *KRT5-CreER*^*T2*^ mice (tamoxifen-inducible Cre in basal epithelial cells) with Tr*p53*^*fl/fl*^*; Rb*^*fl/fl*^ or *p53*^*fl/fl*^; *Ink4a/Arf*^*fl/fl*^ mice, and applying 4-hydroxytamoxifen and the carcinogen, benzo[a]pyrene, topically to oral mucosa of these genetically engineered mice. Mice containing varying sizes of tumours were administered i.p. with 80 mg/kg N_3_-AZA: pimonidazole in a 1:1 ratio. 2 h after injection, mice were sacrificed, tumours and organs (brain, lung, liver, kidney and cervical lymph nodes) removed and frozen in optimal cutting temperature compound (O.C.T, Fisher Healthcare 4585). Serial sections (7–10 μm each) from tumours and organs were taken, dried and stored at -80 °C.

**Tissue staining:** Frozen sections were air dried for 25 min, fixed in acetone for 5 min precooled to -20 °C, air dried 25 min and blocked in PBS with 1% BSA for 20 min. Sections were incubated with primary antibodies for small vessel endothelium (rat anti-CD31, 1:50 dilution; 550274, BD Pharmingen) and hypoxia (rabbit anti-pimonidazole, 1:100 dilution; Hypoxyprobe) for 2 h at room temperature, followed by three 5-min washes in PBS. The secondary antibody (Alexafluor 647 goat anti-rabbit, 1:1000 dilution; Molecular Probes), with 4',6-diamidino-2-phenylindole (DAPI) to stain DNA, was incubated for 30 min at 37 °C in the dark, followed by three 5-min washes with PBS. The Click-iT reaction cocktail (containing Alexafluor 555-conjugated alkyne) was incubated with the sections for 30 min. Sections were washed 5 times with PBS for 5 min and mounted using Mowiol 4–88 (Millipore, Etobicoke, ON) mounting media. Slides were stored at 4 °C until imaged. Images were obtained by tile scan with a Leica SP8 STED microscope.

**Hypoxia fraction calculation**: An estimate of hypoxic tumour fraction was calculated with MATLAB R2018b (MATLAB and Image Processing Toolbox Release 2018b, The MathWorks, Inc., Natick, Massachusetts, United States) using a modified protocol described previously [24]. To account for background staining on the click channel, a manual threshold was determined from a negative control (click staining performed on tumour section from saline injected mice) and was applied for the N_3_-AZA stained slides. The Otsu threshold was chosen for DAPI [25]. Images were converted to binary according to their respective threshold and the hypoxic fraction estimate was calculated using following formula:

$$\mathrm{Hypoxic}\;\mathrm{fraction}\;(\mathrm{HF})=\frac{\mathrm{Area}\;\mathrm{of}\;\mathrm{pixels}\;\mathrm{stained}\;\mathrm{with}\;N3-\mathrm{AZA}}{\mathrm{Area}\;\mathrm{of}\;\mathrm{pixels}\;\mathrm{stained}\;\mathrm{with}\;\mathrm{DAPI}}\times 100$$

**Data processing and statistics**: Immunoblots and microscopic images were processed with Adobe Photoshop to adjust for brightness and contrast, to add scale bars, and for rearrangement and labelling. Gel scans were quantified using Image Studio lite v5.2 (LI-COR) and normalized to vehicle treated normoxia controls. Microscopic images were processed with IMARIS software (Bitplane, Zürich, Switzerland) to quantify area and channel intensity of cell, nucleus and cytoplasm. GraphPad Prism V7 (GraphPad Software, La Jolla, CA) was used to generate graphs and to perform statistical analysis. Non-liner regression analysis was performed on crystal violet staining data to obtain IC_50_ values. Graphs display the mean with standard error of the mean (S.E.M.). Statistical analysis was performed using 2-tailed unpaired *t*-test, with p < 0.05 considered statistically significant. GAPDH activity was analyzed using Dunnett's 2-way ANOVA. Asterisks depict statistically significant differences: ns (not significant), * (P ≤ 0.05), ** (P ≤ 0.01), *** (P < 0.001), **** (P < 0.0001).

## 3 Results

**Fig. 1:**
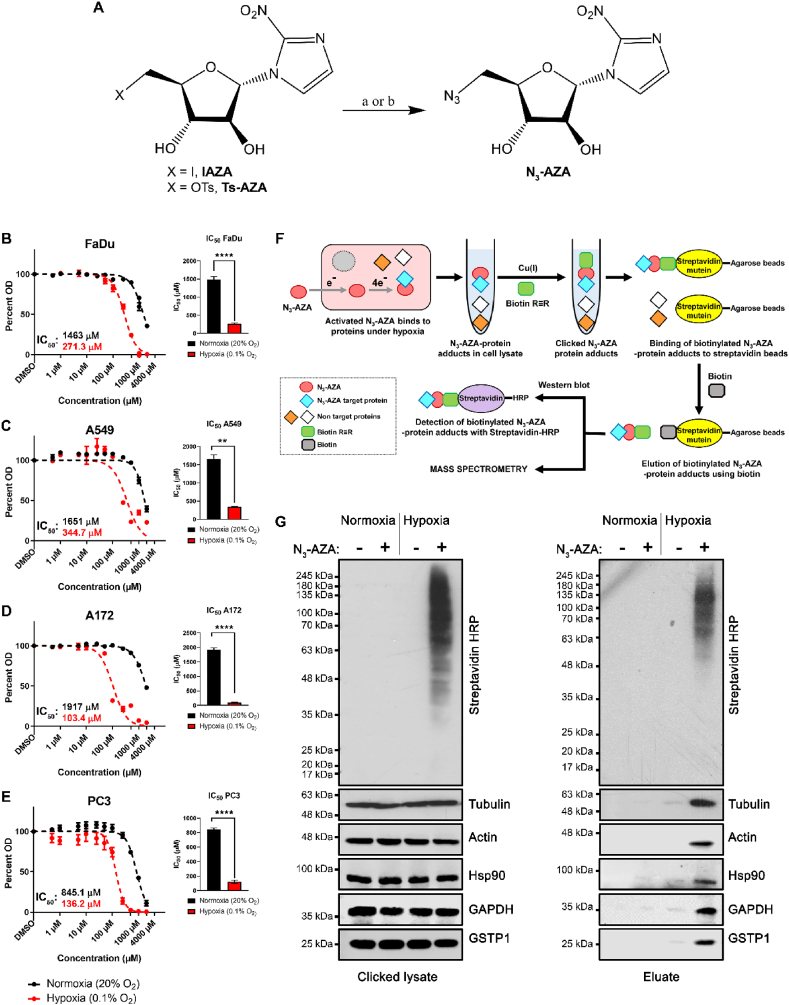
**N**_**3**_**-AZA synthesis, cytotoxicity and click chemistry principle to isolate 2-NI target proteins.** (A) Synthesis of N_3_-AZA. Reagents and conditions: (a) **Ts-AZA**, NaN_3_, DMSO, 50 °C, overnight, 69%; (b) **IAZA**, NaN_3_, DMF, 100 °C, 2 h, 93%. N_3_-AZA shows preferential cytotoxicity in hypoxic FaDu (B), A549 (C), A172 (D) and PC3 cells (E), with statistically significant differences between their normoxic and hypoxic IC_50_ values. Data represents mean ± S.E.M. from at least three independent experiments. (F) Experimental design for isolation and visualization of N_3_-AZA bound proteins. (G) Click chemistry was performed on cell extracts collected from N_3_-AZA (or DMSO) treated normoxic and hypoxic FaDu cells using a biotin alkyne. Western blotting showed that the signal for Streptavidin-HRP is only present in drug treated hypoxic samples. Drug bound proteins could successfully be isolated using streptavidin-mutein beads, with no significant background binding. Representative immunoblots are displayed from three independent experiments.

**Synthesis of N**_**3**_**-AZA**: Fig. 1A depicts the synthesis of the target compound N_3_-AZA from two precursors namely Ts-AZA [17] and IAZA [18]. Since the latter route provided better scalability and no detectable side products, the reaction was carried out on a multigram scale with a yield of 93%. Atomic numbering and NMR analysis of N_3_-AZA is shown in Fig. S1. N_3_-AZA dissolves in DMSO and remains active, even in solution, for months when stored at -20 °C.

**N**_**3**_**-AZA is selectively cytotoxic to hypoxic cells:** The hypoxia selectivity of N_3_-AZA was first validated to determine doses that would limit the utility of the compound. A crystal violet staining (CVS) assay was used to determine the cytotoxicity of the drug under normoxia and hypoxia [26]. In all four human cancer cell lines tested, N_3_-AZA was more toxic under hypoxia than normoxia (Fig. 1B–E). Cytotoxicity of N_3_-AZA in FaDu cells was directly compared to that of pimonidazole. For N_3_-AZA, hypoxic FaDu cells had an IC_50_ value > 5 fold lower than under normoxia (normoxic and hypoxic IC_50_ values for N_3_-AZA were ~1463 μM and ~271 μM, respectively). Importantly, the compound showed almost no toxicity at concentrations ≤100 μM, even under hypoxia (Fig. 1B). A similar toxicity profile was seen with pimonidazole, with an IC_50_ of ~179 μM under hypoxia and ~3035 μM under normoxia (Fig. S8).

**Fig. 2:**
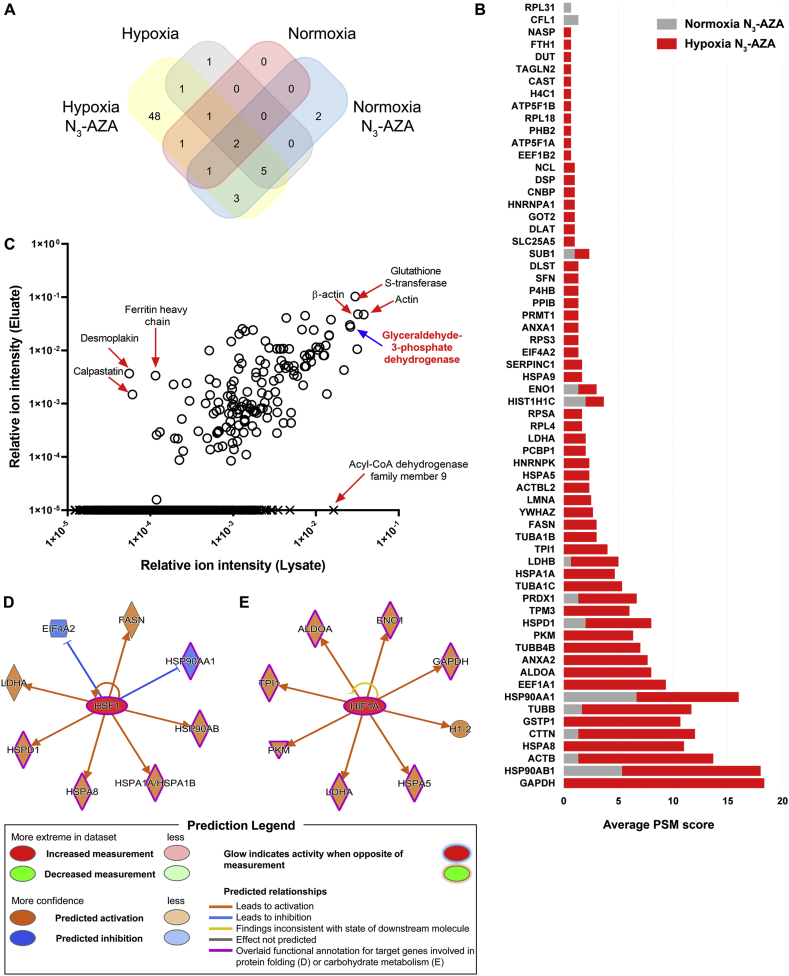
**Mass spectrometric analysis of N**_**3**_**-AZA target proteins.** (A) Venn diagram showing the distribution of proteins identified by mass spectroscopic analysis based on the different treatment conditions. (B) Comparison of PSM values for proteins identified in eluates from N_3_-AZA treated normoxic and hypoxic cells. Data represent cumulative averages for each protein from three independent experiments. (C) Enrichment analysis demonstrated that the likelihood N_3_-AZA labelling is generally dependent on the abundance of target proteins. (D and E) Upstream regulatory analysis by IPA identified 2 clusters of 8 proteins, each under the regulation of a common upstream regulator HSF1 (D) or HIF1A (E). 5 of HSF1 downstream targets are implicated in protein folding while 7 of HIF1A downstream targets are involved in carbohydrate metabolism.

**Identification of proteins that interact with 2-NI under hypoxia:** In hypoxic cells, activated (bioreduced) 2-NIs bind to cellular proteins and form drug-protein adducts. To identify these protein targets, normoxic and hypoxic FaDu cells were treated with N_3_-AZA (or 0.02% DMSO vehicle), lysed, and click chemistry was performed on protein lysates with a biotin labelled alkyne. “Clicked” lysates were processed for western blotting and probed using horse radish peroxidase-conjugated streptavidin (streptavidin-HRP), which showed that “clicked” proteins were present only in N_3_-AZA treated hypoxic cells. These proteins were then successfully isolated with streptavidin-mutein beads (Fig. 1F). Immunoblotting confirmed low background binding of non-biotinylated proteins to the column matrix (Fig. 1G). The recovered proteins were identified by mass spectrometry, and bioinformatic analysis was carried out for functional characterization. From the combined proteomic dataset of eluates obtained from three independent experiments, a total of 65 proteins were identified with a peptide-spectrum match (PSM) score of ≥2 PSM, of which 5 were in “Normoxia + DMSO” eluate (Table S1), 13 were in “Normoxia + N_3_-AZA” eluate (Table S2), 10 were in “Hypoxia + DMSO” eluate (Table S3) and 62 were in “Hypoxia + N_3_-AZA” eluate (Table 1). Overall, 48 proteins were exclusively identified in the “Hypoxia + N_3_-AZA” eluate (Fig. 2A). All proteins identified in the eluates of cells treated with N_3_-AZA (normoxic and hypoxic) are shown with their average PSM scores (Fig. 2B). Enrichment analysis between crude extracts and eluates from N_3_-AZA-treated hypoxic cells revealed that mostly high abundance proteins reacted with the drug. However, several highly abundant proteins also escaped labelling by N_3_-AZA (e.g., acyl-CoA dehydrogenase family member 9, ACAD9). Likewise, several relatively low-abundant proteins, such as ferritin heavy chain, desmoplakin and calpastatin were readily modified by N_3_-AZA (Fig. 2C). Activated 2-NI compounds are known to attack cellular nucleophiles to form adducts. Cysteine (Cys) has often been proposed as the site of attack on proteins [27]. Hence, N_3_-AZA target protein sequences were analyzed to assess any association with their Cys content. Surprisingly, 5 of these proteins did not have any Cys residues whereas 34 proteins had 1-5 Cys, 17 had 6-10 Cys, 3 had 11-15 Cys, and only 3 proteins had >20 Cys residues (Fig. S2A). This prompted analysis of the dataset for the next most nucleophilic amino acid, proline (Pro) [28]. Interestingly, all but one (tropomyosin alpha-3 chain, TPM3) of the N_3_-AZA target proteins contained proline residues, with almost 60% of them containing >15 Pro residues (Fig. S2B). TPM3, on the other hand, contains 1 Cys residue.

**Table 1:** Proteins identified in eluates from N_3_-AZA treated hypoxic cells using LC-MS/MS.

Gene	Protein name	Average PSM score	Size (amino acid)	#Cys	#Pro
GAPDH	Glyceraldehyde-3-phosphate dehydrogenase	18.33	335	3	12
HSP90AB1	Heat shock protein HSP 90-beta	12.67	724	6	23
ACTB	Actin, cytoplasmic 1	12.33	375	6	19
HSPA8^a^	Heat shock cognate 71 kDa protein	11.00	646	4	25
CTTN	Src substrate cortactin	10.67	550	3	17
GSTP1^a^	Glutathione S-transferase P	10.67	210	4	11
TUBB	Tubulin beta chain	10.00	444	8	20
HSP90AA1	Heat shock protein HSP 90-alpha	9.33	732	7	21
EEF1A1^a^	Elongation factor 1-alpha 1	9.33	462	6	25
ALDOA^a^	Fructose-bisphosphate aldolase A	8.00	364	8	19
ANXA2^a^	Annexin A2	7.67	339	4	7
TUBB4B^a^	Tubulin beta-4B chain	7.00	445	8	20
PKM^a^	Pyruvate kinase PKM	6.33	531	10	24
HSPD1	60 kDa heat shock protein, mitochondrial	6.00	573	3	19
TPM3^a^	Tropomyosin alpha-3 chain	6.00	285	1	0
PRDX1	Peroxiredoxin-1	5.33	199	4	13
TUBA1C^a^	Tubulin alpha-1C chain	5.33	449	12	20
HSPA1A^a^	Heat shock 70 kDa protein 1A	4.67	641	5	24
LDHB	l-lactate dehydrogenase B chain	4.33	334	5	11
TPI1	Triosephosphate isomerase	4.00	286	5	10
TUBA1B^a^	Tubulin alpha-1B chain	3.00	451	12	20
FASN^a^	Fatty acid synthase	3.00	2511	46	149
YWHAZ	14-3-3 protein zeta/delta	2.67	245	3	4
LMNA^a^	Prelamin-A/C	2.50	664	5	15
ACTBL2^a^	Beta-actin-like protein 2	2.33	376	6	21
HSPA5^a^	Endoplasmic reticulum chaperone BiP	2.33	654	2	27
HNRNPK^a^	Heterogeneous nuclear ribonucleoprotein K	2.33	463	5	41
PCBP1^a^	Poly(rC)-binding protein 1	2.00	356	9	19
LDHA^a^	l-lactate dehydrogenase A chain	2.00	332	5	11
RPL4^a^	60S ribosomal protein L4	1.67	427	5	25
RPSA^a^	40S ribosomal protein SA	1.67	295	2	20
HIST1H1C	Histone H1.2	1.67	213	0	21
ENO1	Alpha-enolase	1.67	434	6	16
HSPA9^a^	Stress-70 protein, mitochondrial	1.67	679	5	22
SERPINC1^a^	Antithrombin-III	1.67	464	8	21
EIF4A2^a^	Eukaryotic initiation factor 4A-II	1.33	407	4	12
RPS3^a^	40S ribosomal protein S3	1.33	243	3	17
ANXA1^a^	Annexin A1	1.33	346	4	8
PRMT1^a^	Protein arginine N-methyltransferase 1	1.33	371	11	12
PPIB^a^	Peptidyl-prolyl cis-trans isomerase B	1.33	216	1	8
P4HB^a^	Protein disulfide-isomerase	1.33	508	7	21
SFN^a^	14-3-3 protein sigma	1.33	248	2	7
DLST^a^	Dihydrolipoyllysine-residue succinyltransferase component of 2-oxoglutarate dehydrogenase complex, mitochondrial	1.33	453	6	39
SUB1	Activated RNA polymerase II transcriptional coactivator p15	1.33	127	0	6
SLC25A5^a^	ADP/ATP translocase 2	1.00	298	4	7
DLAT^a^	Acetyltransferase component of pyruvate dehydrogenase complex	1.00	647	9	69
GOT2^a^	Aspartate aminotransferase, mitochondrial	1.00	430	7	21
HNRNPA1^a^	Heterogeneous nuclear ribonucleoprotein A1	1.00	372	2	10
CNBP^a^	Cellular nucleic acid-binding protein	1.00	177	22	4
DSP^a^	Desmoplakin	1.00	2871	43	55
NCL^a^	Nucleolin	1.00	710	1	31
EEF1B2^a^	Elongation factor 1-beta	0.67	225	3	10
ATP5F1A^a^	ATP synthase subunit alpha, mitochondrial	0.67	553	2	18
PHB2^a^	Prohibitin	0.67	299	0	10
RPL18^a^	60S ribosomal protein L18	0.67	188	2	10
ATP5F1B^a^	ATP synthase subunit beta	0.67	529	0	32
H4C1^a^	Histone H4	0.67	103	0	1
CAST^a^	Calpastatin	0.67	708	7	69
TAGLN2^a^	Transgelin-2	0.67	199	3	9
DUT^a^	Deoxyuridine 5'-triphosphate nucleotidohydrolase, mitochondrial	0.67	252	5	21
FTH1^a^	Ferritin heavy chain	0.67	183	3	3
NASP^a^	Nuclear autoantigenic sperm protein	0.67	788	5	33

a Proteins identified exclusively in this group.

The 62 putative target proteins of N_3_-AZA were categorized using PANTHER classification system (cellular component, molecular function and biological process) (Fig. S2C). Cellular component analysis showed that N_3_-AZA target proteins are mostly cytoplasmic (44 out of 62 proteins; ~71%), followed by 13 nuclear proteins (~21%), 4 plasma membrane proteins and 1 with an annotated extracellular matrix localization. According to molecular function, most of the proteins belong to the subcategories of binding (33), catalytic activity (24) and structural activity (12). Biological process-related proteins mainly belong to the subcategories of cellular process, metabolic process, localization, biological regulation and response to stimulus. IPA identified literature reported canonical pathways these proteins are involved in, and the top 10 pathways (based on the P value) are listed (Table 2). The dataset was also analyzed to assess if these target proteins are under the regulation of common upstream regulators. Two clusters, pertinent to hypoxic response, are reported here: (i) cluster 1 regulated by heat shock transcription factor 1, HSF1 (8 proteins) (Fig. 2D) and (ii) cluster 2 regulated by hypoxia inducible factor 1 subunit alpha, HIF1A (8 proteins) (Fig. 2E).

**Table 2:** Top 10 canonical pathways identified through IPA analysis.

Ingenuity Canonical Pathways	P-value^a^		% Overlap^b^	Molecules
BAG2 Signaling Pathway		1.81E-09	14.0% (6/43)	ANXA2, HSP90AA1, HSPA1A, HSPA5, HSPA8, HSPA9
Glycolysis I		8.05E-09	19.2% (5/26)	ALDOA, ENO1, GAPDH, PKM, TPI1
HIF1A Signaling		8.48E-08	3.9% (8/205)	HSP90AA1, HSPA1A, HSPA5, HSPA8, HSPA9, LDHA, LDHB, PKM
Sirtuin Signaling Pathway		9.12E-08	3.1% (9/291)	ATP5F1A, ATP5F1B, GOT2, H1-2, LDHA, LDHB, SLC25A5, TUBA1B, TUBA1C
EIF2 Signaling		1.68E-07	3.6% (8/224)	ACTB, EIF4A2, HNRNPA1, HSPA5, RPL18, RPL4, RPS3, RPSA
Aldosterone Signaling in Epithelial Cells		2.44E-07	4.4% (7/158)	HSP90AA1, HSP90AB1, HSPA1A, HSPA5, HSPA8, HSPA9, HSPD1
Unfolded protein response		4.39E-07	8.9% (5/56)	HSPA1A, HSPA5, HSPA8, HSPA9, P4HB
Role of PKR in Interferon Induction and Antiviral Response		7.93E-07	5.1% (6/117)	HSP90AA1, HSP90AB1, HSPA1A, HSPA5, HSPA8, HSPA9
Remodeling of Epithelial Adherens Junctions		1.17E-06	7.4% (5/68)	ACTB, TUBA1B, TUBA1C, TUBB, TUBB4B
14-3-3-mediated Signaling		1.28E-06	4.7% (6/127)	SFN, TUBA1B, TUBA1C, TUBB, TUBB4B, YWHAZ

a P-value: p-value of overlap calculated using Fisher's Exact Test.

b % Overlap: number of proteins from our dataset overlapped with total proteins in a certain pathway in IPA knowledgebase.

**Fig. 3:**
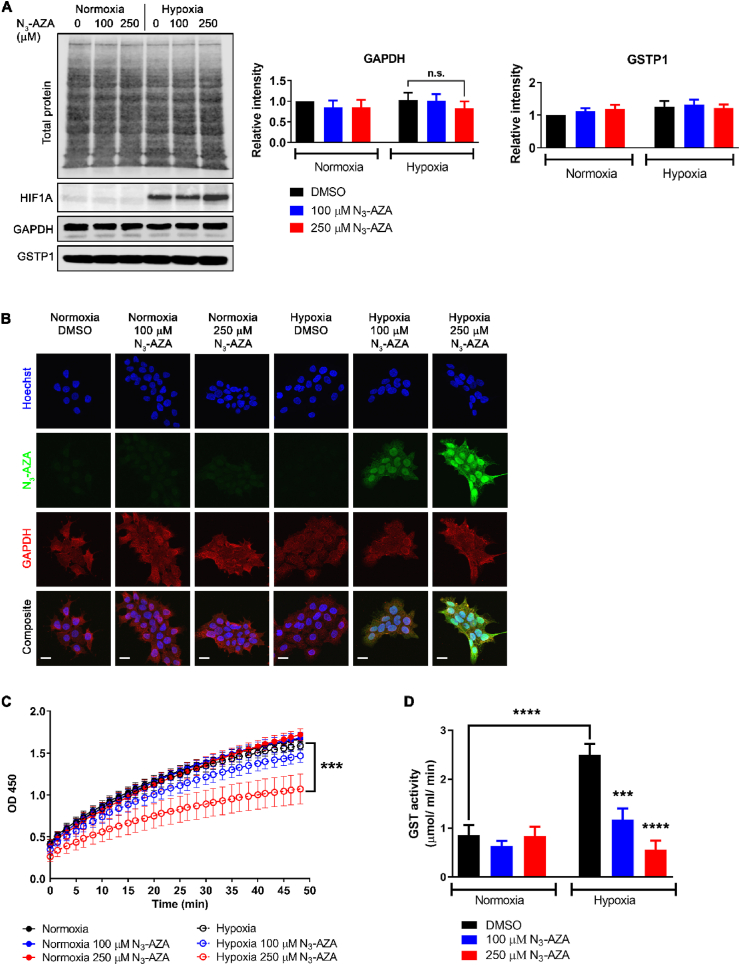
**Effects of N_3_-AZA on GAPDH and GSTP1 protein levels, GAPDH localization and their enzymatic activity.** (A) Lysates prepared from N_3_-AZA (or 0.02% DMSO) treated normoxic and hypoxic FaDu cells were processed for western blotting. N_3_-AZA treatment did not alter GAPDH and GSTP1 protein levels regardless of O_2_ conditions. Representative immunoblots and quantitation [mean ± S.E.M.] from three independent experiments are displayed. (B) Cells treated with N_3_-AZA (or 0.02% DMSO) under normoxia and hypoxia were processed for immunocytochemistry to monitor GAPDH localization; no change in cellular localization of GAPDH was observed in response to N_3_-AZA treatment. The micrographs are representative of at least three independent experiments; scale bar = 20 μm. (C and D) FaDu cells treated with N_3_-AZA (or 0.02% DMSO) under normoxia and hypoxia were processed for GAPDH activity assay (C) or GST activity assay (D); the enzymatic activities of GAPDH and GST were significantly reduced only in N_3_-AZA treated hypoxic cells. Data represent mean ± S.E.M. from three independent experiments.

**N**_**3**_**-AZA treatment did not affect the levels and localization of target proteins, but reduced GAPDH and GSTP1 enzymatic activity under hypoxia:** To assess the effects of N_3_-AZA treatment on its target proteins, the following proteins were studied: actin, tubulin, heat shock protein 90 (Hsp-90), glyceraldehyde-3-phosphate dehydrogenase (GAPDH) and glutathione S-transferase P (GSTP1); HIF1A was used as an indicator of successful hypoxia induction. Hypoxic cells treated with 250 μM N_3_-AZA appeared more compact, with fewer projections, however no significant difference was observed in target protein levels nor their cellular localization following N_3_-AZA treatment (Fig. 3A and B; Fig. S3). To examine if N_3_-AZA treatment affects GAPDH enzymatic activity, we used an assay that measures NADH generation during GAPDH catalyzed oxidation of glyceraldehyde-3-phosphate (GAP) into 1,3-bisphosphoglycerate (BPG); the amount of NADH generated is proportional to the activity of GAPDH present during the reaction time. N_3_-AZA, at a concentration close to its hypoxic IC_50_ value (250 μM), significantly reduced GAPDH enzyme activity only under hypoxia (0.1254 nmol/min/μL vs 0.08511 nmol/min/μL in 0.02% DMSO and 250 μM N_3_-AZA treated samples, respectively). No effect on GAPDH activity was seen under normoxia (~0.13 nmol/min/μL in all treatment groups) (Fig. 3C). Total GST activity was determined using an assay that measures the conjugation of 1-chloro-2,4-dinitrobenzene (CDNB) with reduced glutathione, resulting in an increase in absorbance at 340 nm. The rate of increase is directly proportional to the GST activity in the sample. While hypoxic exposure markedly increased GST activity in vehicle treated cells, in agreement with others [29], N_3_-AZA treated hypoxic cells showed a significant dose-dependent reduction in GST activity (Fig. 3D). We estimate a minimum of 40 ± 13% GAPDH and 57 ± 5% GSTP1 protein was modified by cell exposure to 100 μM N_3_-AZA (Figs. S4–5).

**Fig. 4:**
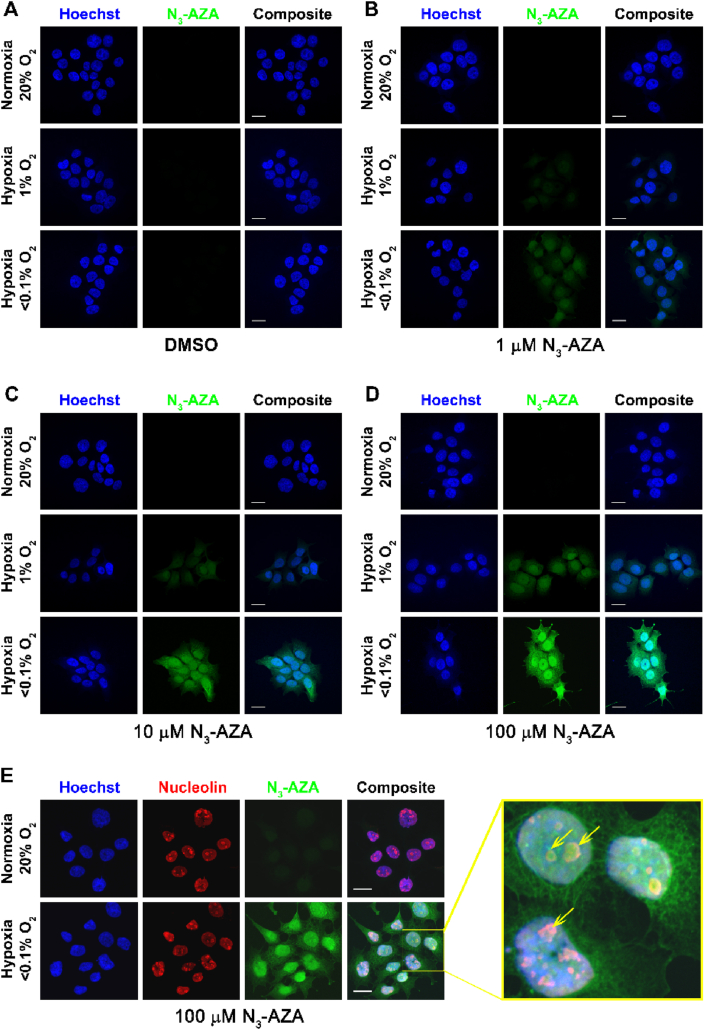
**N**_**3**_**-AZA click chemistry as a hypoxia marker.** (A–D) FaDu cells, treated with different concentration of N_3_-AZA (or 0.02% DMSO vehicle control) were incubated under normoxia or hypoxia (0.1% O_2_ or <0.1% O_2_), and click chemistry was performed on paraformaldehyde fixed cells using a fluorescently tagged alkyne. N_3_-AZA click staining was present only in drug treated hypoxic cells. Intensity of N_3_-AZA click staining increased with drug concentration and decreased with O_2_ levels. (E) N_3_-AZA click staining is concentrated in nucleoli. Micrographs displayed are representative of at least three independent experiments; scale bar = 20 μm.

**Sub-cellular localization of N**_**3**_**-AZA:** Click chemistry between the azido moiety of N_3_-AZA and a fluorophore-conjugated alkyne enabled us to image the sub-cellular localization of N_3_-AZA adducts formed under hypoxia using fluorescence microscopy. Fluorescent staining was observed only in O_2_-starved cells when treated with N_3_-AZA for 6 h (Fig. 4A–D); incubation duration was determined from a separate time course experiment (Fig. S6A). Furthermore, the compound was entrapped in FaDu cells in an inverse oxygen-dependent manner, with the highest retention seen in cells cultured under <0.1% O_2_. Normoxic cells showed minimal to almost no click staining. Staining of N_3_-AZA under hypoxia increased in a dose dependent manner, with 100 μM concentration demonstrating the most vivid differences (Fig. 4D, Fig. S6B). N_3_-AZA-treated hypoxic cells showed strong nuclear staining (Fig. S6C) with a nucleolar uptake pattern; the latter was confirmed by immunostaining for nucleolin (Fig. 4E, Movie 1). A similar hypoxia selective N_3_-AZA staining pattern was confirmed in additional cancer cell lines (Fig. S6D).

Supplementary video related to this article can be found at https://doi.org/10.1016/j.redox.2021.101905

The following is the supplementary data related to this article:

Multimedia component 1 Multimedia component 1

To assess the metabolic stability of N_3_-AZA-protein adducts upon reoxygenation, FaDu cells were treated with 100 μM N_3_-AZA for 6 h under hypoxia and then reoxygenated, fixed at the indicated time points and processed for N_3_-AZA click staining; N_3_-AZA was present in the medium throughout the reoxygenation period. N_3_-AZA click fluorescent signal could be detected up to 24 h following reoxygenation, suggesting that N_3_-AZA modification is relatively stable. Although the intensity weakened by about 40%, the decrease in intensity appeared to be a consequence of cell doubling (data not shown). Minimal background was seen in their normoxic counterparts even after prolonged incubation with the compound (Fig. S7).

**Fig. 5:**
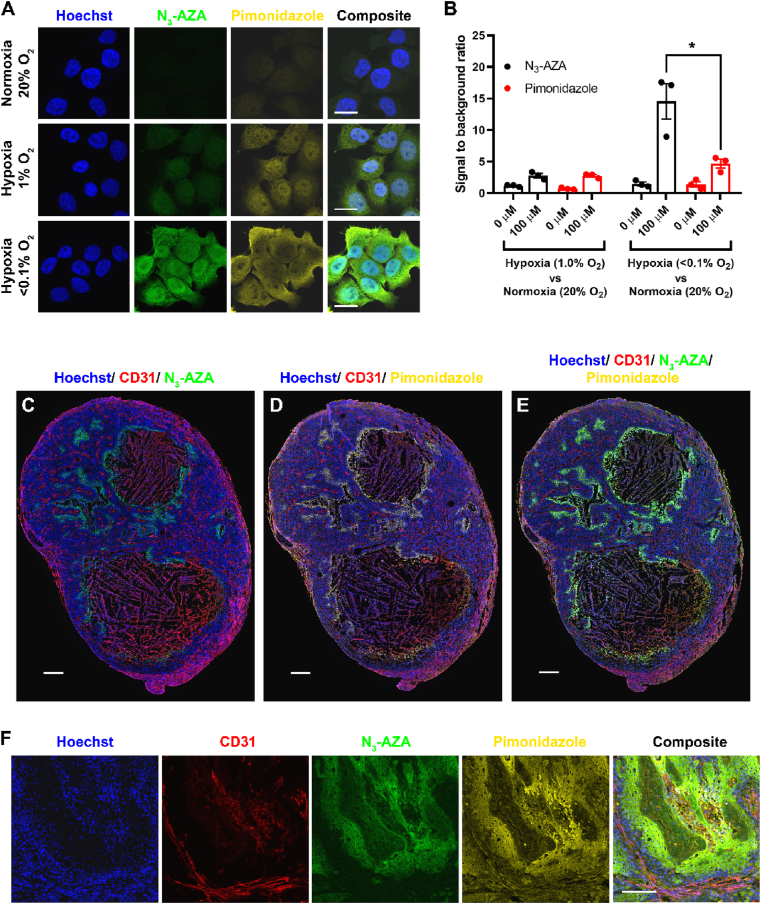
**Pimonidazole immunostaining is comparable to that of N**_**3**_**-AZA.** (A) Representative micrographs from three independent experiments showing that N_3_-AZA click staining and pimonidazole immunostaining overlaps in hypoxic FaDu cells co-treated with both compounds. (B) The micrographs were processed with IMARIS software to quantify channel intensities from N_3_-AZA click staining and pimonidazole immunostaining. The ratios of signal (hypoxia):background (normoxia) intensities for cells co-treated with N_3_-AZA and pimonidazole [or vehicle control (0.02% DMSO) i.e. columns labelled 0 μM, see Fig. S7 for micrographs of cells not treated with N_3_-AZA or pimonidazole] are shown (mean ± S.E.M.). N_3_-AZA click staining generated a higher signal to noise ratio compared to pimonidazole immunostaining. (C–F) *In vivo* comparison of N_3_-AZA click staining with pimonidazole immunostaining. Both are concentrated in the same regions of a mouse subcutaneous tumour section (C–E) and of a primary mouse head and neck tumour section (F). Representative micrographs are displayed from at least three independent experiments; scale bar represents 20 μm (A), 1 mm (C–E) and 200 μm (F).

To confirm the hypoxia selectivity of N_3_-AZA click staining, cells treated simultaneously with pimonidazole and N_3_-AZA were processed for pimonidazole immunostaining followed by N_3_-AZA click chemistry. Both N_3_-AZA click staining and pimonidazole immunostaining were detected only in drug treated hypoxic cells, with minimal signal in normoxic samples (Fig. 5A). Both staining strategies were equally efficient at differentiating between normoxic and hypoxic cells, however, N_3_-AZA click staining proved to be simpler and faster compared to pimonidazole immunostaining and produced better signal to background ratio (Fig. 5B). Interestingly, although both compounds contain 2-NI moieties, they showed perceptibly different cellular staining patterns. N_3_-AZA generated a strong nucleolar signal in hypoxic cells (Movie 1), whereas pimonidazole staining remained excluded from nucleoli (Movie 2).

Supplementary video related to this article can be found at https://doi.org/10.1016/j.redox.2021.101905

The following is the supplementary data related to this article:

Multimedia component 2 Multimedia component 2

**N**_**3**_**-AZA click chemistry for hypoxia mapping in tumours:** Since N_3_-AZA click chemistry could efficiently stain hypoxic cells, its capacity to detect hypoxic regions in tumour tissue was examined in comparison to pimonidazole. Initially, a FaDu cell subcutaneous xenograft model was used, which has previously been shown to have a hypoxic fraction of 11–12% [30]. Regions of tumour hypoxia defined by the click chemistry reaction were present in areas bordering necrosis and were independent of the vasculature (Fig. 5C). Pimonidazole immunostaining generated a similar staining pattern (Fig. 5D). Pimonidazole and N_3_-AZA staining co-localized to areas bordering necrotic regions as expected (Fig. 5E). These findings were then validated in mouse primary head and neck tumours (Fig. 5F). For both tumour models, a low level of non-specific staining of tumour sections by the secondary antibodies used for pimonidazole and CD31 was observed; N_3_-AZA click staining provided a clearer background. We further quantified the hypoxic fraction in tumours with different volumes to determine if there is a relationship between tumour size and hypoxia. Our data (Fig. S10A) did not indicate a size dependence, which implies that small tumours may retain a hypoxic fraction that is resistant to therapy. Importantly, significant click staining was not seen in any of the harvested organs, which suggests that N_3_-AZA preferentially accumulates in hypoxic tumour niches (Figs. S10B–G).

## 4 Discussion

The tumour microenvironment has garnered considerable attention in recent times for its role in therapy outcome [31]. Hypoxia, a key feature of the tumour microenvironment, offers both a challenge and a vulnerability that can be exploited for therapy. However, very few strategies have yielded successful results when it comes to targeting hypoxic tumours. Despite several setbacks, NIs still hold significant value as hypoxia directed therapeutics. For example, nimorazole (a 5-NI), in combination with radiotherapy, showed promise in treating advanced head and neck cancer patients [32]. Interestingly, meta analysis of NI clinical trials showed an overall improvement in patient outcome, particularly when stratified depending on tumour oxygenation status [33]. Therefore, an in depth understanding of NI molecular mechanism is crucial to better guide future development of NI based cancer therapeutics.

NIs have long been known to bind to nucleic acids and proteins in hypoxic cells [34]. While early NI studies almost exclusively concentrated on their potential to induce DNA damage [[35], [36], [37]], NI protein targets remained relatively unexplored. A handful of proteomic studies do exist, albeit mostly limited to reaction of 5-NIs in protozoans [[38], [39], [40]]. Recently, a click chemistry based method to identify candidate 5-NI drug targets in *Giardia lamblia* using a modified metronidazole (metronidazole alkyne) and an azido-biotin was outlined [41]. The current study shows that N_3_-AZA click chemistry with a biotin-alkyne is compatible with mammalian cell-based assays to efficiently biotinylate proteins that interact with activated 2-NIs under hypoxia (Fig. 1G). These proteins were then isolated, identified by mass spectrometry and characterized for functional significance with respect to molecular targeting of hypoxia. We report a total of 62 protein targets of N_3_-AZA, 48 of which were not present in our control samples (Fig. 2A, Table 1). For proteins that appeared ubiquitously across the panel, their abundance was generally multiple-fold higher in the N_3_-AZA treated hypoxic sample (based on PSM scores, Fig. 2B). To assess if N_3_-AZA is binding to a subset of proteins, a relative enrichment profile was generated by comparing crude extracts and eluates from N_3_-AZA treated hypoxic cells (Fig. 2C). Overall, N_3_-AZA-protein interaction appeared to be dictated by the abundance of the protein, with a few exceptions; several highly abundant proteins (such as ACAD9) did not react with N_3_-AZA, while proteins like desmoplakin and calpastatin, despite being relatively low in abundance, appeared as positive hits. This suggests that there might be some degree of selectivity for NI-protein interaction. In contrast to previous reports proposing thiol groups as a major site for NI attack [27], no clear association was found between the Cys content of a protein and its likelihood of binding to N_3_-AZA, which is consistent with the findings described by Ref. [41]. For example, despite having 11 Cys residues, ACAD9 escaped N_3_-AZA binding while 5 of the N_3_-AZA target proteins (histone H1.2, prohibitin, ATP synthase subunit beta, histone H4 and activated RNA polymerase II transcriptional coactivator p15) lack any Cys residues (Fig. S2A). While this observation could be a result of Cys residues being buried within the protein structure or forming cysteine-cysteine disulfide bonds, it could also imply that other nucleophilic amino acids, such as Pro, may serve as the site of adduct formation for NI compounds. Indeed, the current analysis showed that most of the N_3_-AZA target proteins are enriched for Pro residues (Fig. S2B). Unfortunately, without the exact chemical formula of the N_3_-AZA-amino acid derivatives we were unable to identify potential conjugates by MS.

Analysis of the proteomic data was performed using several bioinformatics platforms. N_3_-AZA target proteins appeared to be predominantly cytoplasmic (~71%) with only ~21% being nuclear proteins (Fig. S2C). Upstream regulatory analysis with IPA identified two regulatory clusters with direct implications to hypoxic response. Cluster 1 (regulated by HSF1) has 8 downstream targets, 5 of which are molecular chaperones directly involved in adaptations to low O_2_ levels and protect against reoxygenation-induced oxidative stress (HSP90AA1, HSP90AB1, HSPA1A, HSPA8 and HSPD1) (Fig. 2D) [42,43]. Of particular interest to us was cluster 2, consisting of 8 target proteins of N_3_-AZA, which are under the regulation of HIF1A. HIF1A, known as the master regulator of hypoxia, plays a key role in cellular response to hypoxic insults [44]. Hypoxia induced stabilization of HIF1A results in upregulation of pro-survival genes, including those involved in glucose metabolism. 7 out of the 8 N_3_-AZA target proteins in cluster 2 are implicated in carbohydrate metabolism (Fig. 2E). Whether or not binding of N_3_-AZA (and other 2-nitoimidazole compounds) to these downstream targets can counteract the actions of HIF1A remains to be explored.

Interestingly, from our list of 62 target proteins (Table 1), canonical pathway analysis identified glycolysis I as one of the top hits with a P value of 8.05E-09; five of the N_3_-AZA target proteins belong to this pathway (ALDOA, ENO1, GAPDH, PKM and TPI1) and are regulated by HIF1A (Table 2, Fig. 2E). It is important to note that reprogramming of glucose metabolism through upregulation of glycolysis is a crucial hallmark of tumourigenesis. Despite being less efficient than the mitochondrial respiration system, cancer cells readily utilize aerobic glycolysis for ATP production, mostly to counteract mitochondrial defects or to compensate for the defective mitochondrial oxidative phosphorylation under hypoxia. Increased glycolysis also provides an acidic microenvironment through elevated production of lactate, and thus facilitates malignant progression [45]. GAPDH was identified as the top target for N_3_-AZA with an average PSM score of 18.3. It is a Cys-directed glycolytic enzyme that catalyzes the reversible conversion of glyceraldehyde-3-phosphate (GAP) to 1,3-bisphosphoglycerate (BPG). GAPDH transcription has been reported to be upregulated in hypoxic cells in a HIF1A dependent manner [46,47], however, such regulation appears to be cell-type specific [48]. In addition to its role in glycolysis, GAPDH has also been implicated in diverse physiological and pathophysiological processes [45,49,50]. This makes GAPDH an interesting target to follow-up on. Three aspects of N_3_-AZA treatment on GAPDH were explored: total protein level, cellular distribution and enzymatic activity. Total GAPDH protein levels remained unaffected in response to N_3_-AZA treatment, both under normoxia and hypoxia. (Fig. 3A). NIs (such as PA-824) can induce nitrosative stress by acting as an NO donor, which can lead to oxidization/S-nitrosylation of GAPDH and its translocation to the nucleus [51]. Immunocytochemical analysis showed that N_3_-AZA treatment did not alter GAPDH cellular distribution (Fig. 3B). The highly conserved active site of GAPDH contains a Cys residue, which is readily acetylated during the reversible oxidative phosphorylation of GAP [52], is essential for its role in oxidative stress response [[53], [54], [55]], and could potentially serve as a binding site to 2-NIs. Importantly, ornidazole (a 5-NI) has been reported to inhibit GAPDH activity in mouse spermatocytes [56], protozoa and anaerobic bacteria [57], and depletion of GAPDH has also been reported to induce cytostatic effects on human cancer cell lines [58]. This prompted us to explore the effects of N_3_-AZA treatment on GAPDH enzymatic activity. GAPDH activity did not differ significantly between the normoxic-treatment groups, whereas upon hypoxic incubation, its enzymatic activity was reduced by >30% in cells treated with 250 μM N_3_-AZA as compared to vehicle control (Fig. 3C).

Another key protein target identified in our analysis was GST family protein GSTP1 (Table 1), which is the predominant non-hepatic isozyme that eliminates toxic by-products of oxidative stress and xenobiotics by catalyzing their conjugation with glutathione (GSH) [59]. GSTP1 has also been implicated in the mitogen-activated protein (MAP) kinase pathway through protein: protein interactions with c-Jun N-terminal kinase 1 (JNK1) and apoptosis signal-regulating kinase (ASK1), which are activated in response to cellular stress [60]. While GSTs can prevent tumour formation by protecting cellular proteins and DNA from endogenous reactive compounds, their detoxification properties may aid in multi-drug resistance in tumour cells. Indeed, tumours often express high levels of GST (particularly GSTP1), and GSTs are frequently overexpressed in multidrug-resistant cells [61]. Additionally, hypoxic exposure is often accompanied by an increase in GST activity to cope with the altered intracellular redox potential and the generation of increased cellular reactive oxygen species (ROS) [29]. Interestingly, inhibition of GSTP1 reduces cell viability and induces vulnerability to both intracellular oxidative stress and hypoxia/reoxygenation stress [62]. Nitroimidazoles, such as etanidazole and misonidazole, have previously been reported to inhibit GST activity [63,64]. Our analysis is in agreement with these findings, and show a significant reduction in GST specific activity only in N_3_-AZA treated hypoxic cells. Compared to vehicle treated cells, we observed >50% reduction in hypoxic cells treated with 100 μM N_3_-AZA and >75% reduction in hypoxic cells treated with 250 μM N_3_-AZA (Fig. 3D). The inhibitory effect of the drug does not seem to be due to proteolysis since no significant change in GSTP1 protein levels was observed in response to drug treatment or hypoxic exposure (Fig. 3A). While our data does not preclude toxicity arising from DNA damage [35], given the dynamic roles of GAPDH and GSTP1 in the hypoxic tumour microenvironment, such an effect of N_3_-AZA on their activities may partially explain the observed hypoxic cytotoxicity of the drug.

The list of NI-targeted proteins (Table 1) includes additional proteins that demand further attention, for example, peroxiredoxin-1 (Prdx1). Similar to other members of this protein family, Prdx1 active site contains two Cys residues that are essential for its catalytic function and could serve as potential binding sites for thiol-reacting NIs [65]. It is therefore tempting to postulate that binding of N_3_-AZA to Prdx1 through its catalytic Cys residues may exert a negative impact on its enzymatic activity. Prdx1 plays a key role in cellular detoxification, functions as a chaperone, has anti-apoptotic property, is upregulated in cancer and contributes to radioresistance [66,67]. Interestingly, Prdx1 is upregulated under hypoxia possibly to counteract the detrimental effects of hypoxia/reoxygenation mediated accumulation of reactive oxygen species and to provide aggressive survival advantage for malignant progression [66,68]. Hence, N_3_-AZA binding and subsequent reduction in enzymatic activity of Prdx1 could provide an additional mechanism for its hypoxic cytotoxicity. Further studies will be required to test this hypothesis. Interestingly, a related protein, thioredoxin reductase (TrxR), has previously been reported as a common target for 5-NIs across three microorganisms [[38], [39], [40]].

A unique feature of N_3_-AZA click chemistry is its versatility since it can also be used as a hypoxia marker. N_3-_AZA shares structural homology with ^18^F-FAZA, which itself has proven to be superior in comparison to other 2-NI hypoxia radiotracers [69]. 2-NIs are widely used for immunohistological hypoxia diagnosis, aided by the development of antibodies against pimonidazole- and EF-5-protein adducts [15,16]. N_3_-AZA click chemistry offers a simpler alternative by exploiting the Cu(I) catalyzed click chemistry instead to visualize cellular hypoxia. While the 2-NI moiety of N_3_-AZA ensures that it is selectively forming drug-protein adducts in hypoxic cells, the azido group can be exploited to fluorescently label these adducts using a fluorescently tagged alkyne molecule in the presence of a Cu(I) catalyst. A similar strategy for fluorescent labelling of hypoxic cells using an alkyne conjugated NI, SN33267, has previously been reported in a patent and a MSc. thesis [70,71].

N_3_-AZA click fluorescence is extremely selective for hypoxic cells, with minimal background staining in their normoxic counterparts. It significantly reduces the staining duration (30 min versus 2.5 h for pimonidazole) while still maintaining high fidelity. An inverse relationship between O_2_ levels and N_3_-AZA click staining intensity is reported (Fig. 4A–D, S4B). N_3_-AZA demonstrated preferential uptake by the nucleus (Fig. S6C) and concentrated in nucleoli (Fig. 4E, Movie 1). While this does not necessarily agree with the proteomic analysis (N_3_-AZA target proteins being predominantly cytoplasmic, Fig. S2C), some of the nuclear N_3_-AZA click staining may be attributed to the formation of drug-nucleic acid adducts. N_3_-AZA-nucleic acid binding was not investigated here, however, previous studies reported direct binding of activated NIs to DNA [34].

N_3_-AZA and pimonidazole showed similar cytotoxicity patterns in FaDu cells (preferential toxicity under hypoxia) (Fig. 1B & **S8**). Both pimonidazole immunostaining and N_3_-AZA click chemistry efficiently labelled hypoxic cells, however, the latter was significantly faster and produced a clearer background (Fig. 5A and B). The efficiency of N_3_-AZA and pimonidazole as hypoxia probes was also compared *in vivo*, using two mouse models of head and neck cancer; (i) FaDu cell based subcutaneous tumour model (Fig. 5C–E) and (ii) a Cre-LoxP-based primary mouse model of HPV-negative squamous cell carcinoma (Fig. 5F). In tumour sections, N_3_-AZA- and pimonidazole-stained areas overlapped, suggesting that N_3_-AZA click chemistry is staining the same regions that are considered hypoxic by pimonidazole immunostaining (Fig. 5E). As expected, both N_3_-AZA and pimonidazole stained regions were mostly mutually exclusive with vasculature (CD31 positive regions). N_3_-AZA click chemistry following pimonidazole immunostaining did not affect the latter (Fig. 5D). Also, no significant click staining was detected in organs (brain, lung, liver, kidney and cervical lymph nodes) harvested from tumour-bearing mice administered with N_3_-AZA, indicating that N_3_-AZA is preferentially accumulating in the hypoxic regions of tumours (Figs. S10B–G). An important clinical question is to what degree does tumour size dictate hypoxia levels? Previous data obtained by computerized Eppendorf pO_2_ histography indicated that hypoxia is independent of tumour size [72]. Our data with N_3_-AZA click staining performed on tumours ranging in volume between ~150 and 350 mm^3^ also indicated no clear association between tumour size and hypoxia levels (Fig. S10A).

In summary, N_3_-AZA click chemistry provided a robust chemical strategy to identify potential protein targets of 2-NI compounds in a human head and neck tumour model. N_3_-AZA binding to the glycolytic enzyme GAPDH and detoxification enzyme GSTP1 showed a profound effect on their enzymatic activities only under hypoxia, which creates an interesting avenue for not only further mechanistic exploration of NI compounds in general, but also suggests their potential role in targeted hypoxia therapy. In addition, the modularity of the N_3_-AZA click reaction allowed for its application as an antibody independent histological hypoxia marker that offers a faster but equally efficient alternative to pimonidazole immunostaining.

## Declaration of competing interest

The authors Michael Weinfeld, Piyush Kumar and Hassan El-Saidi are included in the following patent application: Markers, conjugates, compositions and methods for hypoxia imaging, mapping, and therapy. International PCT Patent, Application No. PCT/CA2018/051165. Other authors declare no competing interests.

## References

[1] Bertout J.A., Patel S.A., Simon M.C. (2008). The impact of O 2 availability on human cancer. Nat. Rev. Canc..

[2] Janssen H., Haustermans K., Balm A., Begg A. (2005). Hypoxia in head and neck cancer: how much, how important?. Head Neck.

[3] Zeng M., Kikuchi H., Pino M.S., Chung D.C. (2010). Hypoxia activates the K-ras proto-oncogene to stimulate angiogenesis and inhibit apoptosis in colon cancer cells. PLOS One.

[4] House S.W., Warburg O., Burk D., Schade A.L. (1956). On respiratory impairment in cancer cells. Science.

[5] Kumareswaran R., Ludkovski O., Meng A., Sykes J., Pintilie M., Bristow R.G. (2012). Chronic hypoxia compromises repair of DNA double-strand breaks to drive genetic instability. J. Cell Sci..

[6] Teicher B.A. (1995). Angiogenesis and cancer metastases: therapeutic approaches. Crit. Rev. Oncol.-Hematol..

[7] Keith B., Simon M.C. (2007). Hypoxia-inducible factors, stem cells, and cancer. Cell.

[8] Walsh J.C., Lebedev A., Aten E., Madsen K., Marciano L., Kolb H.C. (2014). The clinical importance of assessing tumor hypoxia: relationship of tumor hypoxia to prognosis and therapeutic opportunities. Antioxidants Redox Signal..

[9] Ai M., Budhani P., Sheng J., Balasubramanyam S., Bartkowiak T., Jaiswal A.R., Ager C.R., Haria D.D., Curran M.A. (2015). Tumor hypoxia drives immune suppression and immunotherapy resistance. J. Immunother. Canc..

[10] Kizaka‐Kondoh S., Konse‐Nagasawa H. (2009). Significance of nitroimidazole compounds and hypoxia‐inducible factor‐1 for imaging tumor hypoxia. Canc. Sci..

[11] Overgaard J., Hansen H.S., Andersen A., Hjelm-Hansen M., Jørgensen K., Sandberg E., Berthelsen A., Hammer R., Pedersen M. (1989). Misonidazole combined with split-course radiotherapy in the treatment of invasive carcinoma of larynx and pharynx: report from the DAHANCA 2 study. Int. J. Radiat. Oncol. Biol. Phys..

[12] Lee D.-J., Cosmatos D., Marcial V.A., Fu K.K., Rotman M., Cooper J.S., Ortiz H.G., Beitler J.J., Abrams R.A., Curran W.J. (1995). Results of an RTOG phase III trial (RTOG 85-27) comparing radiotherapy plus etanidazole with radiotherapy alone for locally advanced head and neck carcinomas. Int. J. Radiat. Oncol. Biol. Phys..

[13] Rajendran J., Wilson D., Conrad E., Peterson L., Bruckner J., Rasey J., Chin L., Hofstrand P., Grierson J., Eary J. (2003). [18 F] FMISO and [18 F] FDG PET imaging in soft tissue sarcomas: correlation of hypoxia, metabolism and VEGF expression. Eur. J. Nucl. Med. Mol. Imag..

[14] Mortensen L.S., Johansen J., Kallehauge J., Primdahl H., Busk M., Lassen P., Alsner J., Sørensen B.S., Toustrup K., Jakobsen S. (2012). FAZA PET/CT hypoxia imaging in patients with squamous cell carcinoma of the head and neck treated with radiotherapy: results from the DAHANCA 24 trial. Radiother. Oncol..

[15] Varia M.A., Calkins-Adams D.P., Rinker L.H., Kennedy A.S., Novotny D.B., Fowler W.C., Raleigh J.A. (1998). Pimonidazole: a novel hypoxia marker for complementary study of tumor hypoxia and cell proliferation in cervical carcinoma. Gynecol. Oncol..

[16] Evans S.M., Hahn S., Pook D.R., Jenkins W.T., Chalian A.A., Zhang P., Stevens C., Weber R., Weinstein G., Benjamin I., Mirza N., Morgan M., Rubin S., Mckenna W.G., Lord E.M., Koch C.J. (2000). Detection of hypoxia in human squamous cell carcinoma by EF5 binding. Canc. Res..

[17] Kumar P., Wiebe L., Asikoglu M., Tandon M., Mcewan A. (2002). Microwave-assisted (radio) halogenation of nitroimidazole-based hypoxia markers. Appl. Radiat. Isot..

[18] Mannan R.H., Somayaji V.V., Lee J., Mercer J.R., Chapman J.D., Wiebe L.I. (1991). arabinofuranosyl)-2-thtroimidazole. J. Nucl. Med..

[19] Koch C., Howell R., Biaglow J. (1979). Ascorbate anion potentiates cytotoxicity of nitro-aromatic compounds under hypoxic and anoxic conditions. Br. J. Canc..

[20] Pathan M., Keerthikumar S., Ang C.S., Gangoda L., Quek C.Y., Williamson N.A., Mouradov D., Sieber O.M., Simpson R.J., Salim A. (2015). FunRich: an open access standalone functional enrichment and interaction network analysis tool. Proteomics.

[21] Mi H., Muruganujan A., Ebert D., Huang X., Thomas P.D. (2019). Panther version 14: more genomes, a new PANTHER GO-slim and improvements in enrichment analysis tools. Nucleic Acids Res..

[22] Kramer A., Green J., Pollard J., Tugendreich S. (2014). Causal analysis approaches in ingenuity pathway analysis. Bioinformatics.

[23] Mowery Y.M., Carpenter D.J., Wisdom A.J., Badea C.T., Luo L., Ma Y., Pendse A., Kirsch D.G. (2018). Characterization of novel primary mouse models of HPV-negative squamous cell carcinoma of the oral cavity. Radiation Research Society Annual Meeting. Chicago, Illinois.

[24] Dubois L.J., Lieuwes N.G., Janssen M.H., Peeters W.J., Windhorst A.D., Walsh J.C., Kolb H.C., Öllers M.C., Bussink J., Van Dongen G.A. (2011). Preclinical evaluation and validation of [18F] HX4, a promising hypoxia marker for PET imaging. Proc. Natl. Acad. Sci. Unit. States Am..

[25] Otsu N. (1979). A threshold selection method from gray-level histograms. IEEE Trans. Syst. Man Cybernet..

[26] Feoktistova M., Geserick P., Leverkus M. (2016). Crystal violet assay for determining viability of cultured cells. Cold Spring Harb. Protoc..

[27] Wislocki P.G., Bagan E.S., Vandenheuvel W.J., Walker R.W., Alvaro R.F., Arison B.H., Lu A.Y., Wolf F.J. (1984). Drug residue formation from ronidazole, a 5-nitroimidazole. V. Cysteine adducts formed upon reduction of ronidazole by dithionite or rat liver enzymes in the presence of cysteine. Chem. Biol. Interact..

[28] Brotzel F., Mayr H. (2007). Nucleophilicities of amino acids and peptides. Org. Biomol. Chem..

[29] Millar T.M., Phan V., Tibbles L.A. (2007). ROS generation in endothelial hypoxia and reoxygenation stimulates MAP kinase signaling and kinase-dependent neutrophil recruitment. Free Radic. Biol. Med..

[30] Yaromina A., Zips D., Thames H.D., Eicheler W., Krause M., Rosner A., Haase M., Petersen C., Raleigh J.A., Quennet V. (2006). Pimonidazole labelling and response to fractionated irradiation of five human squamous cell carcinoma (hSCC) lines in nude mice: the need for a multivariate approach in biomarker studies. Radiother. Oncol..

[31] Hirata E., Sahai E. (2017). Tumor microenvironment and differential responses to therapy. Cold Spring Harb. Perspect. Med..

[32] Overgaard J., Hansen H.S., Overgaard M., Bastholt L., Berthelsen A., Specht L., Lindeløv B., Jørgensen K. (1998). A randomized double-blind phase III study of nimorazole as a hypoxic radiosensitizer of primary radiotherapy in supraglottic larynx and pharynx carcinoma. Results of the Danish Head and Neck Cancer Study (DAHANCA) Protocol 5-85. Radiother. Oncol..

[33] Overgaard J. (1994). Clinical evaluation of nitroimidazoles as modifiers of hypoxia in solid tumors. Oncol. Res..

[34] Varghese A., Whitmore G. (1980). Binding to cellular macromolecules as a possible mechanism for the cytotoxicity of misonidazole. Canc. Res..

[35] Edwards D.I., Alexander P., Gielen J., Sartorelli Ac (2013). Reduction of nitroimidazoles in vitro and DNA damage. Bioreduction in the Activation of Drugs.

[36] Zahoor A., Lafleur M., Knight R., Loman H., Edwards D. (1987). Dna damage induced by reduced nitroimidazole drugs. Biochem. Pharmacol..

[37] Jenner T.J., Sapora O., O'neill P., Fielden E.M. (1988). Enhancement of Dna damage in mammalian cells upon bioreduction of the nitroimidazole-aziridines RSU-1069 and RSU-1131. Biochem. Pharmacol..

[38] Leitsch D., Kolarich D., Wilson I.B., Altmann F., Duchêne M. (2007). Nitroimidazole action in Entamoeba histolytica: a central role for thioredoxin reductase. PLoS Biol..

[39] Leitsch D., Kolarich D., Binder M., Stadlmann J., Altmann F., Duchêne M. (2009). Trichomonas vaginalis: metronidazole and other nitroimidazole drugs are reduced by the flavin enzyme thioredoxin reductase and disrupt the cellular redox system. Implications for nitroimidazole toxicity and resistance. Mol. Microbiol..

[40] Leitsch D., Schlosser S., Burgess A., Duchêne M. (2012). Nitroimidazole drugs vary in their mode of action in the human parasite Giardia lamblia. Int. J. Parasitol.: Drugs Drug Resist..

[41] Lauwaet T., Miyamoto Y., Ihara S., Le C., Kalisiak J., Korthals K.A., Ghassemian M., Smith D.K., Sharpless K.B., Fokin V.V. (2020). Click chemistry-facilitated comprehensive identification of proteins adducted by antimicrobial 5-nitroimidazoles for discovery of alternative drug targets against giardiasis. PLoS Neglected Trop. Dis..

[42] Baird N.A., Turnbull D.W., Johnson E.A. (2006). Induction of the heat shock pathway during hypoxia requires regulation of heat shock factor by hypoxia-inducible factor-1. J. Biol. Chem..

[43] Jain K., Suryakumar G., Ganju L., Singh S.B. (2014). Differential hypoxic tolerance is mediated by activation of heat shock response and nitric oxide pathway. Cell Stress Chaperones.

[44] Nagao A., Kobayashi M., Koyasu S., Chow C.C., Harada H. (2019). HIF-1-dependent reprogramming of glucose metabolic pathway of cancer cells and its therapeutic significance. Int. J. Mol. Sci..

[45] Pelicano H., Martin D., Xu R., Huang P. (2006). Glycolysis inhibition for anticancer treatment. Oncogene.

[46] Yamaji R., Fujita K., Takahashi S., Yoneda H., Nagao K., Masuda W., Naito M., Tsuruo T., Miyatake K., Inui H. (2003). Hypoxia up-regulates glyceraldehyde-3-phosphate dehydrogenase in mouse brain capillary endothelial cells: involvement of Na+/Ca2+ exchanger. Biochim. Biophys. Acta Mol. Cell Res..

[47] Higashimura Y., Nakajima Y., Yamaji R., Harada N., Shibasaki F., Nakano Y., Inui H. (2011). Up-regulation of glyceraldehyde-3-phosphate dehydrogenase gene expression by HIF-1 activity depending on Sp1 in hypoxic breast cancer cells. Arch. Biochem. Biophys..

[48] Said H.M., Hagemann C., Stojic J., Schoemig B., Vince G.H., Flentje M., Roosen K., Vordermark D. (2007). GAPDH is not regulated in human glioblastoma under hypoxic conditions. BMC Mol. Biol..

[49] Ganapathy-Kanniappan S., Geschwind J.-F.H. (2013). Tumor glycolysis as a target for cancer therapy: progress and prospects. Mol. Canc..

[50] Tristan C., Shahani N., Sedlak T.W., Sawa A. (2011). The diverse functions of GAPDH: views from different subcellular compartments. Cell. Signal..

[51] Manjunatha U., Boshoff H.I., Barry C.E. (2009). The mechanism of action of PA-824: novel insights from transcriptional profiling. Commun. Integr. Biol..

[52] Knight R.J., Kofoed K.F., Schelbert H.R., Buxton D.B. (1996). Inhibition of glyceraldehyde-3-phosphate dehydrogenase in post-ischaemic myocardium. Cardiovasc. Res..

[53] Hildebrandt T., Knuesting J., Berndt C., Morgan B., Scheibe R. (2015). Cytosolic thiol switches regulating basic cellular functions: GAPDH as an information hub?. Biol. Chem..

[54] Nakajima H., Amano W., Fujita A., Fukuhara A., Azuma Y.-T., Hata F., Inui T., Takeuchi T. (2007). The active site cysteine of the proapoptotic protein glyceraldehyde-3-phosphate dehydrogenase is essential in oxidative stress-induced aggregation and cell death. J. Biol. Chem..

[55] Kubo T., Nakajima H., Nakatsuji M., Itakura M., Kaneshige A., Azuma Y.-T., Inui T., Takeuchi T. (2016). Active site cysteine-null glyceraldehyde-3-phosphate dehydrogenase (GAPDH) rescues nitric oxide-induced cell death. Nitric Oxide.

[56] Bone, Cooper (2000). In vitro inhibition of rat cauda epididymal sperm glycolytic enzymes by ornidazole, α-chlorohydrin and 1-chloro-3-hydroxypropanone. Int. J. Androl..

[57] Marcus Y., Tal N., Ronen M., Carmieli R., Gurevitz M. (2016). The drug ornidazole inhibits photosynthesis in a different mechanism described for protozoa and anaerobic bacteria. Biochem. J..

[58] Phadke M.S., Krynetskaia N.F., Mishra A.K., Krynetskiy E. (2009). Glyceraldehyde 3-phosphate dehydrogenase depletion induces cell cycle arrest and resistance to antimetabolites in human carcinoma cell lines. J. Pharmacol. Exp. Therapeut..

[59] Chatterjee A., Gupta S. (2018). The multifaceted role of glutathione S-transferases in cancer. Canc. Lett..

[60] Townsend D.M., Tew K.D. (2003). The role of glutathione-S-transferase in anti-cancer drug resistance. Oncogene.

[61] Tew K.D. (1994). Glutathione-associated enzymes in anticancer drug resistance. Canc. Res..

[62] Fletcher M.E., Boshier P.R., Wakabayashi K., Keun H.C., Smolenski R.T., Kirkham P.A., Adcock I.M., Barton P.J., Takata M., Marczin N. (2015). Influence of glutathione-S-transferase (GST) inhibition on lung epithelial cell injury: role of oxidative stress and metabolism. Am. J. Physiol. Lung Cell Mol. Physiol..

[63] O'dwyer P., Lacreta F., Walczak J., Cox T., Litwin S., Hoffman J., Zimny M., Comis R. (1993). Phase I/pharmacokinetic/biochemical study of the nitroimadazole hypoxic cell sensitiser SR2508 (etanidazole) in combination with cyclophosphamide. Br. J. Canc..

[64] Kumar K.S., Weiss J.F. (1986). Inhibition of glutathione peroxidase and glutathione transferase in mouse liver by misonidazole. Biochem. Pharmacol..

[65] Hanschmann E.-M., Godoy J.R., Berndt C., Hudemann C., Lillig C.H. (2013). Thioredoxins, glutaredoxins, and peroxiredoxins—molecular mechanisms and health significance: from cofactors to antioxidants to redox signaling. Antioxidants Redox Signal..

[66] Kim Y.-J., Ahn J.-Y., Liang P., Ip C., Zhang Y., Park Y.-M. (2007). Human prx1 gene is a target of Nrf2 and is up-regulated by hypoxia/reoxygenation: implication to tumor biology. Canc. Res..

[67] Ding C., Fan X., Wu G. (2017). Peroxiredoxin 1–an antioxidant enzyme in cancer. J. Cell Mol. Med..

[68] Zhang M., Hou M., Ge L., Miao C., Zhang J., Jing X., Shi N., Chen T., Tang X. (2014). Induction of peroxiredoxin 1 by hypoxia regulates heme oxygenase-1 via NF-κB in oral cancer. PLOS One.

[69] Reischl G., Dorow D.S., Cullinane C., Katsifis A., Roselt P., Binns D., Hicks R.J. (2007). Imaging of tumor hypoxia with [124i] IAZA in comparison with [18F] FMISO and [18F] FAZA–first small animal PET results. J. Pharm. Pharmaceut. Sci..

[70] Tercel M., Pruijn F.B. (2011). Agents and methods for detection and/or imaging of hypoxia. Google Patents.

[71] Hou A.L. (2016). A novel click chemistry-based method to detect hypoxic tumour cells and characterise their gene expression. Researchspace@ Auckland.

[72] Vaupel P., Höckel M., Mayer A. (2007). Detection and characterization of tumor hypoxia using pO2 histography. Antioxidants Redox Signal..

