# Supplementary material for: Identification of proteins and cellular pathways targeted by 2-nitroimidazole hypoxic cytotoxins
Source: Redox Biol. 2021 Feb 21;41:101905. doi: 10.1016/j.redox.2021.101905 (PMC7933538; doi:10.1016/j.redox.2021.101905)
Supplement: Multimedia component 3 [file mmc3.docx]

*Supporting materials*

**Synthesis of N_3_-AZA from Ts-AZA precursor.**

1-α-D-(5-*O*-tosyl-arabinofuranosyl)-2-nitroimidazole (Ts-AZA) (0.028 g, 0.07 mmol) and NaN_3_ (0.0455 g, 0.70 mmol) were dissolved in DMSO (5 mL) and stirred at 50 ºC overnight (16 h). The reaction mixture was quenched with H_2_O (10 mL), allowed to cool to room temperature and the product was extracted in EtOAc (3 x 5 mL). The combined organic extracts were dried over anhydrous Na_2_SO_4_, filtered and solvent was removed under reduced pressure. The crude product was purified using column chromatography (10:1, *v/v*, CH_2_Cl_2_/CH_3_OH) to afford 13 mg (0.048 mmol, 69% yield) of N_3_-AZA.

| **Table S1.** Proteins identified in eluates from DMSO treated normoxic cells using LC-MS/MS | | | | | |
| --- | --- | --- | --- | --- | --- |
| **Gene** | **Protein name** | **PSM score** | **Size**  **(amino acid)** | **#Cys** | **#Pro** |
| HSP90AB1 | Heat shock protein HSP 90-beta | 9 | 724 | 6 | 23 |
| GAPDH | Glyceraldehyde-3-phosphate dehydrogenase | 5 | 335 | 3 | 12 |
| CTTN | Src substrate cortactin | 4 | 550 | 3 | 17 |
| TPI1 | Triosephosphate isomerase | 2 | 286 | 5 | 11 |
| HIST1H1C | Histone H1.2 | 2 | 213 | 0 | 21 |

| **Table S2.** Proteins identified in eluates from N_3_-AZA treated normoxic cells using LC-MS/MS | | | | | |
| --- | --- | --- | --- | --- | --- |
| **Gene** | **Protein name** | **PSM score** | **Size**  **(amino acid)** | **#Cys** | **#Pro** |
| HSP90AA1 | Heat shock protein HSP 90-alpha | 6.67 | 732 | 7 | 21 |
| HSP90AB1 | Heat shock protein HSP 90-beta | 5.33 | 724 | 6 | 23 |
| HIST1H1C | Histone H1.2 | 2.00 | 213 | 0 | 21 |
| HSPD1 | 60 kDa heat shock protein, mitochondrial | 2.00 | 573 | 3 | 19 |
| TUBB | Tubulin beta chain | 1.67 | 444 | 8 | 20 |
| CTTN | Src substrate cortactin | 1.33 | 550 | 3 | 17 |
| CFL1 | Cofilin 1 (Non-muscle), isoform CRA_a | 1.33 | 166 | 4 | 6 |
| ENO1 | Alpha-enolase | 1.33 | 434 | 6 | 16 |
| PRDX1 | Peroxiredoxin-1 (Fragment) | 1.33 | 199 | 4 | 13 |
| ACTB | Actin, cytoplasmic 1 | 1.33 | 375 | 6 | 19 |
| SUB1 | Activated RNA polymerase II transcriptional coactivator p15 | 1.00 | 127 | 0 | 6 |
| LDHB | L-lactate dehydrogenase (Fragment) | 0.67 | 334 | 5 | 11 |
| RPL31 | 60S ribosomal protein L31 | 0.67 | 125 | 0 | 6 |

| **Table S3.** Proteins identified in eluates from DMSO treated hypoxic cells using LC-MS/MS | | | | | |
| --- | --- | --- | --- | --- | --- |
| **Gene** | **Protein name** | **PSM score** | **Size**  **(amino acid)** | **#Cys** | **#Pro** |
| HSP90AB1 | Heat shock protein HSP 90-beta | 10 | 724 | 6 | 23 |
| ENO1 | Alpha-enolase | 10 | 434 | 6 | 16 |
| GAPDH | Glyceraldehyde-3-phosphate dehydrogenase | 7 | 335 | 3 | 12 |
| TUBB | Tubulin beta chain | 6 | 444 | 8 | 20 |
| PRDX1 | Peroxiredoxin-1 (Fragment) | 5 | 199 | 4 | 13 |
| YWHAZ | 14-3-3 protein zeta/delta | 3 | 245 | 3 | 4 |
| ANXA2P2 | Putative annexin A2-like protein | 3 | 339 | 3 | 7 |
| LDHB | L-lactate dehydrogenase (Fragment) | 3 | 334 | 5 | 11 |
| HIST1H1C | Histone H1.2 | 3 | 213 | 0 | 21 |
| HSPD1 | 60 kDa heat shock protein, mitochondrial | 3 | 573 | 3 | 19 |

**
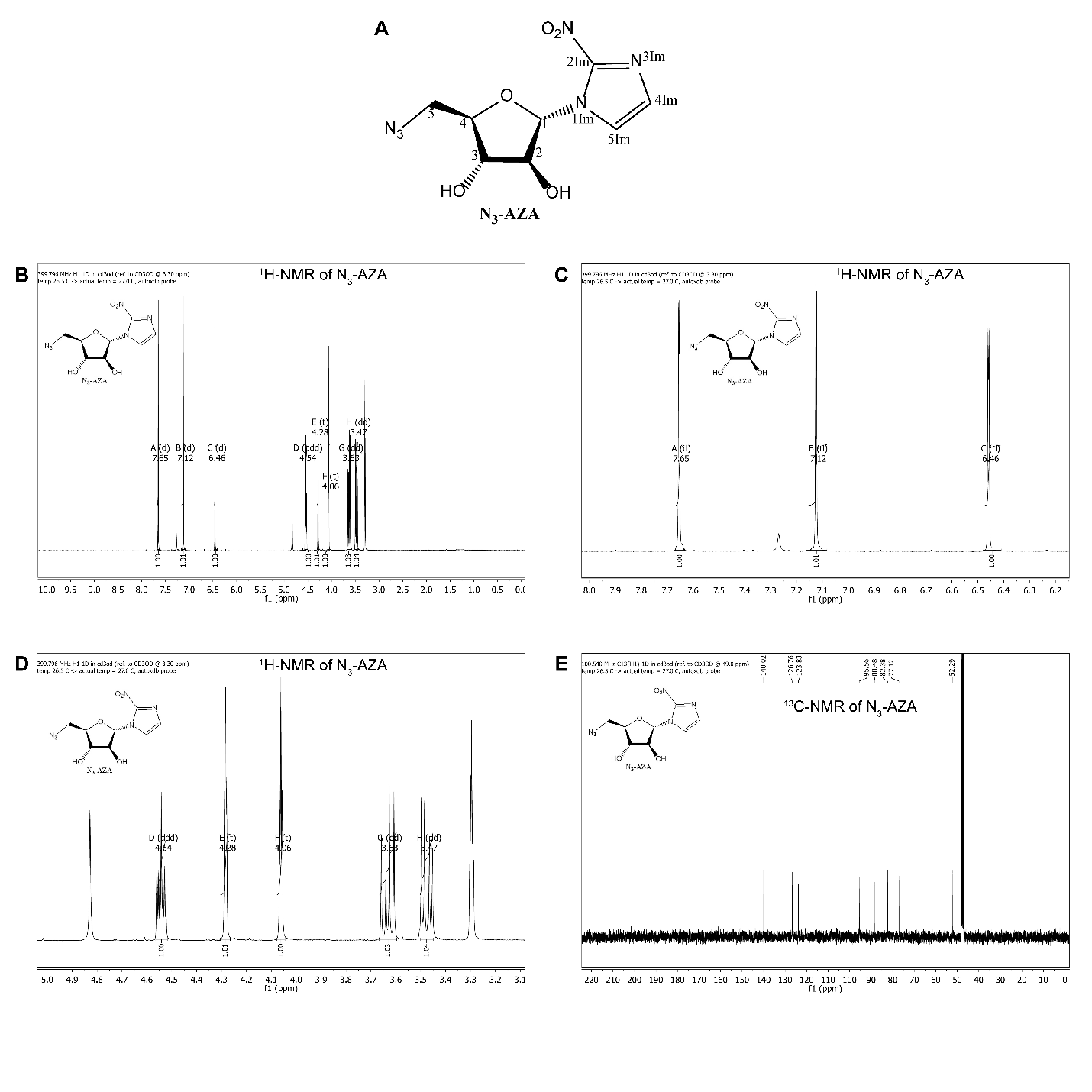
Figure S1. Spectral analysis of N_3_-AZA.** (A) Structure of **N_3_-AZA** and atom assignments used in spectral analysis. (B-D) Proton NMR and (E) carbon-13 NMR of **N_3_-AZA**.


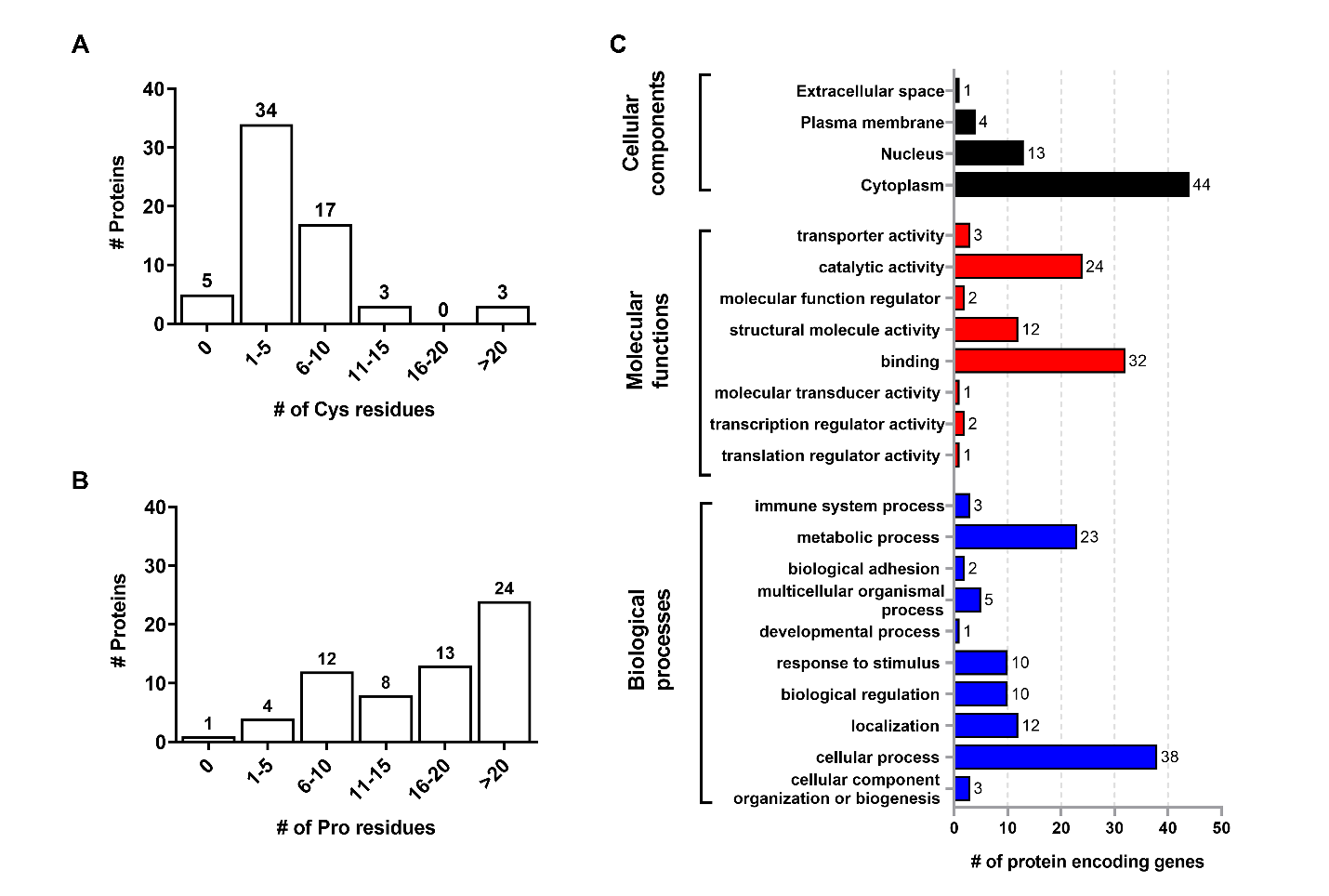


**Figure S2. Functional characterization of N_3_-AZA target proteins.** (A and B) Distribution of N_3_-AZA target proteins based on their cysteine and proline content, respectively. (C) Distribution of N_3_-AZA target proteins based on cellular component, molecular function and biological process, generated by PANTHER classification system.

**
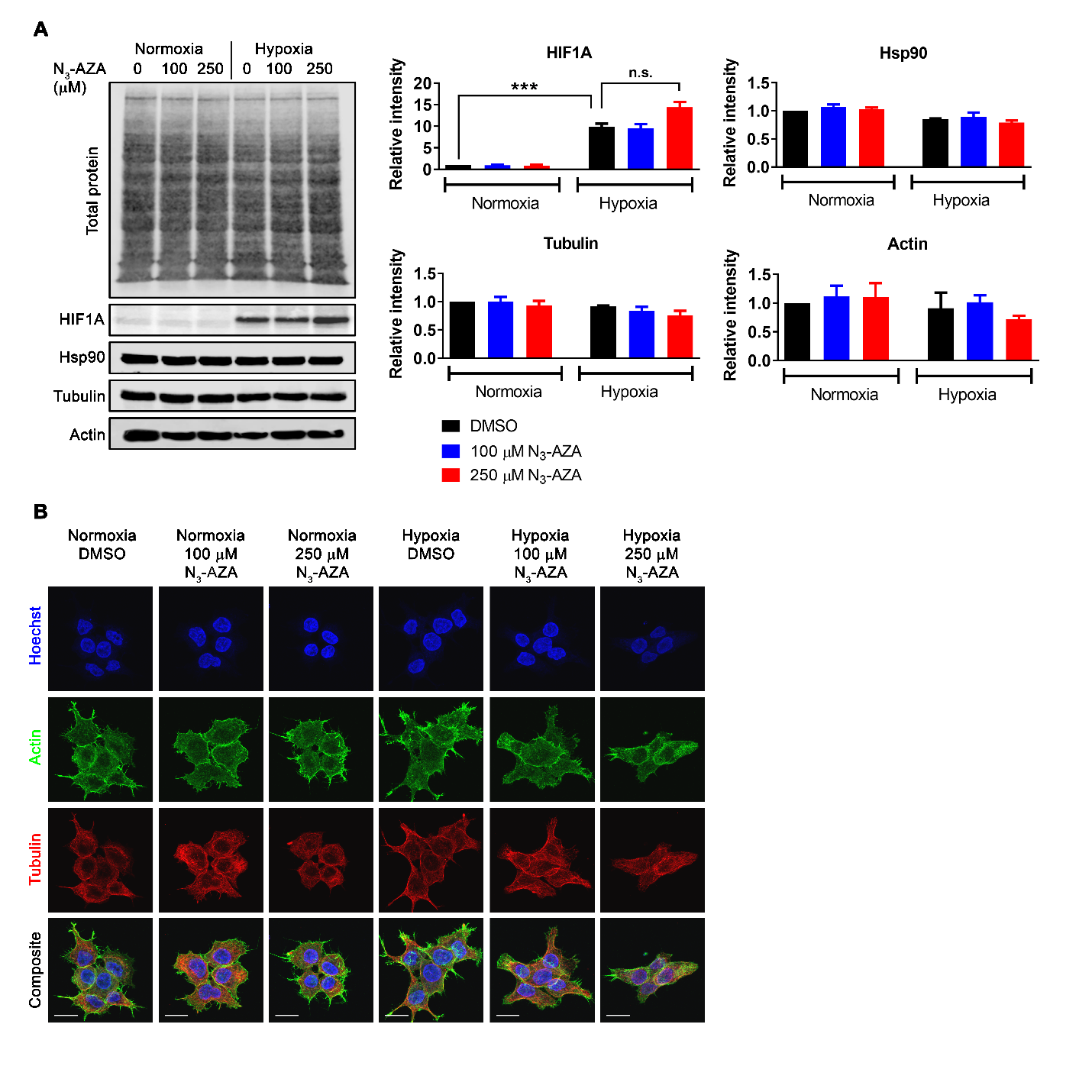
Figure S3**. **Effects of N_3_-AZA treatment on its target proteins.** (A) Extracts were prepared from FaDu cells treated with 0.02% DMSO or N_3_-AZA (100 µM or 250 µM) and processed for HIF1A, Hsp90, tubulin, actin, and total protein by immunoblot. No statistically significant difference was found in target protein levels in response to N_3_-AZA treatment. Representative immunoblots and quantification [mean± S.E.M.] from three independent replicates are displayed. (B) FaDu cells treated with 0.02% DMSO or N_3_-AZA (100 µM or 250 µM) were processed for immunofluorescence imaging for actin (green) and tubulin (blue); nuclei were counter stained with Hoechst. N_3_-AZA treatment did not alter the cellular distribution of these proteins, albeit hypoxic drug treated cells showed fewer cell projections and a condensed morphology. Micrographs presented are representative of at least three independent experiments; scale bar = 20 µm.


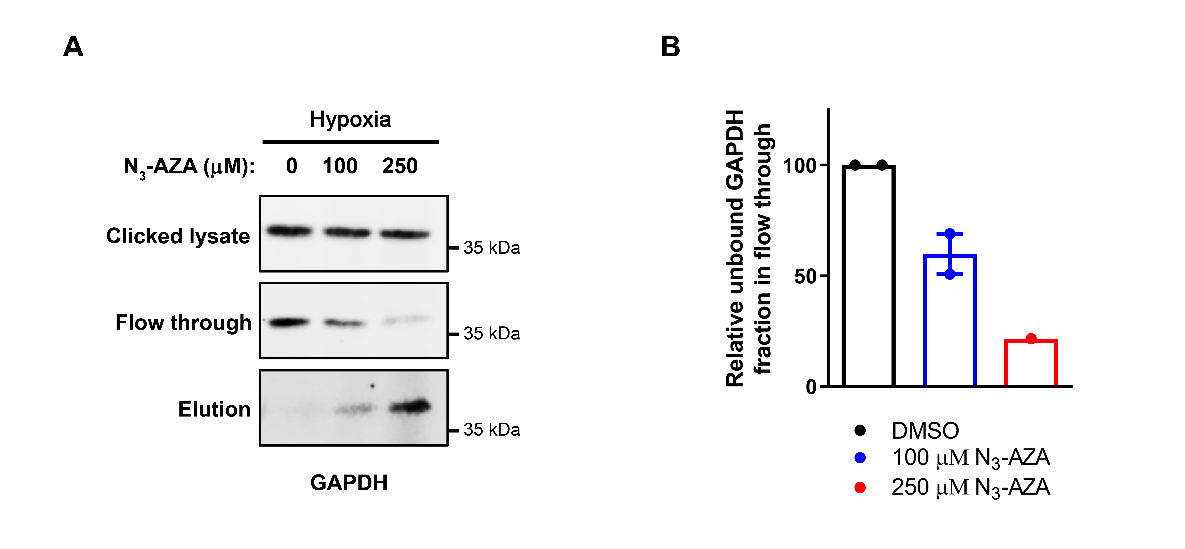


**Figure S4**. **Estimation of N_3_-AZA bound GAPDH fraction.** 300 µg of protein lysates (in 150 µl of RIPA buffer) from hypoxic FaDu cells treated with N_3_-AZA (or vehicle control) were mixed with 150 µl of reaction cocktail containing biotin alkyne for 1 h to allow for click reaction. Clicked lysates were loaded on BSA blocked streptavidin-mutein beads, incubated overnight at 4 ºC, followed by collection of the flowthrough fractions. Beads were resuspended in 300 µl of 1:1 ratio of dH_2_O: SDS loading dye and boiled for 10 min. An equal volume of clicked cell lysates (input), flowthrough fractions (unbound fractions i.e. non-biotinylated proteins) and resuspended beads (bound fractions i.e. biotinylated proteins) were run on SDS-PAGE. Proteins were transferred to nitrocellulose membrane and probed for GAPDH protein (A). GAPDH band intensities in the flowthrough were quantified and normalized to the vehicle treated lane. Relative unbound GAPDH fraction (non-biotinylated i.e. not reacting with N_3_-AZA) in flowthrough is shown in the graph (B). Lane 1 and 2 (DMSO and 100 µM N_3_-AZA treatment) each show the mean± S.E.M. from two independent replicates.

**
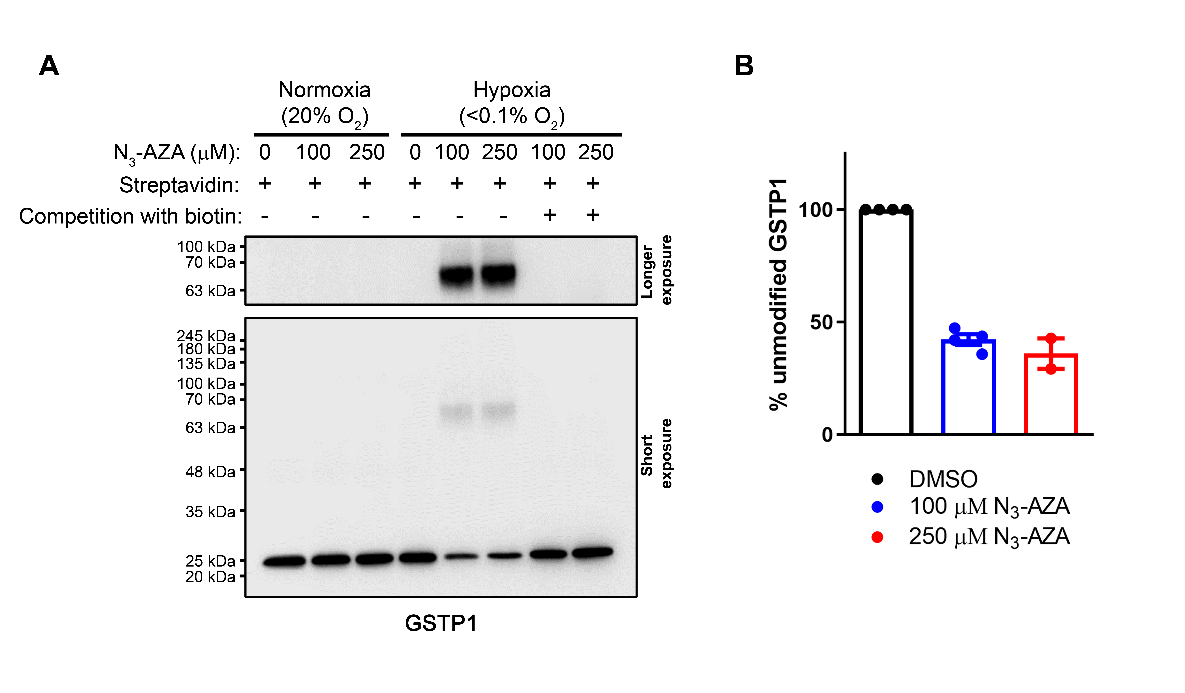
**

**Figure S5**. **Estimation of N_3_-AZA bound GSTP1 fraction.** N_3_-AZA (or vehicle control) treated FaDu cell extracts (normoxic/ hypoxic) underwent reaction with biotin conjugated alkyne for 1 h. Excess biotin from the clicked lysates was removed using microconcentrators (3K cut off, UFC500308, Millipore). Afterwards, the biotin-free clicked lysates (100 µg) were mixed with streptavidin (50 µg, Cat. # 85878, Sigma-Aldrich) and incubated overnight at 4 ºC. For biotin competition samples, 20 µg of biotin was added. Samples were processed for non-denaturing SDS-PAGE and probed for GSTP1. A clear shift in GSTP1 band was seen in hypoxic N_3_-AZA treated clicked samples containing streptavidin. Addition of extra biotin to these samples inhibited this supershift (A). Signal intensities of unmodified GSTP1 were calculated and normalized to the vehicle treated lane. Data show mean± S.E.M. from at least two independent experiments (B).


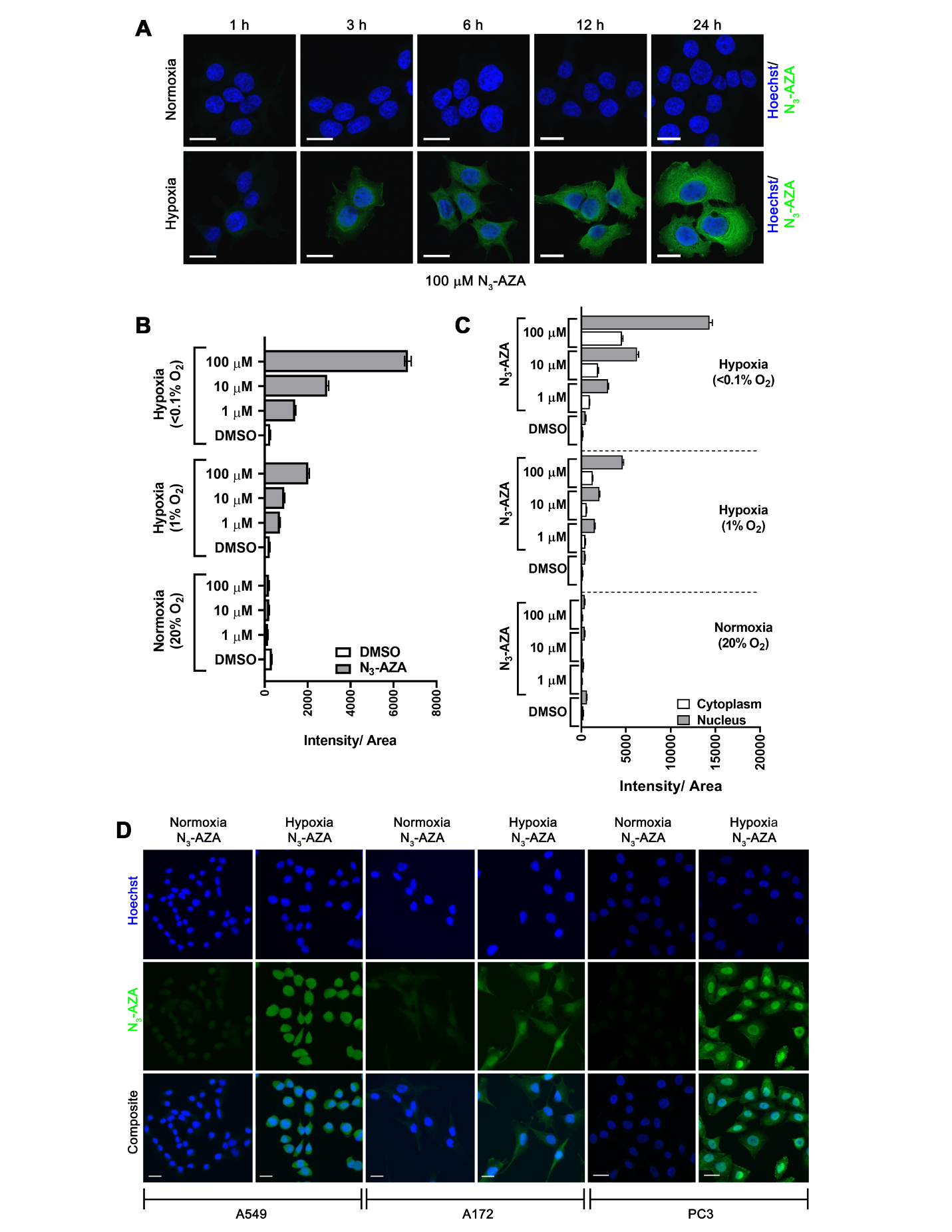


**Figure S6. Intensity of hypoxic N_3_-AZA click staining increases with incubation time, hypoxia levels and is concentrated in the nuclei.** FaDu cells were treated with 100 µM N_3_-AZA for different durations under normoxia (20% O_2_) and hypoxia (<0.1% O_2_), and were fixed in 2% paraformaldehyde at indicated time points. Cells were processed for N_3_-AZA click chemistry and nuclei were counterstained with Hoechst. (A) Confocal microscopy images show that intensity of hypoxia selective N_3_-AZA click staining increased with longer drug incubation, although appreciable intensity was observed in the 6 h treatment group. Notably, no significant N_3_-AZA click staining was observed in normoxic cells, even after prolonged drug incubation. Scale bar= 20 µm. (B) FaDu cells treated with different concentrations of N_3_-AZA (6 h) were processed for N_3_-AZA click chemistry. Confocal microscopy images were processed using IMARIS software to quantify N­_3_-AZA click fluorescence staining intensity along with cell, nuclei and cytoplasm areas. N_3_-AZA click staining is inversely dependent on the O­_2_ levels and is proportional to drug concentration. Quantification shows mean± S.E.M. from three independent replicates. (C) Cell compartment-based analysis identified N_3_-AZA click intensity to be more concentrated in the nucleus. Error bar represents standard error of the mean. (D) A549 lung cancer, A172 glioblastoma and PC-3 prostate cancer cells were treated with 10 µM of N_3_-AZA for 6 h under normoxia (20% O_2_) or hypoxia (<0.1% O_2_), fixed with 2% paraformaldehyde and processed for N_3_-AZA click chemistry and Hoechst staining. Across the panel, hypoxic cells stained positive for N_3_-AZA click staining with minimal staining in their normoxic counterparts. Micrographs are representative of at least two independent experiments; scale bar = 20 µm.

**
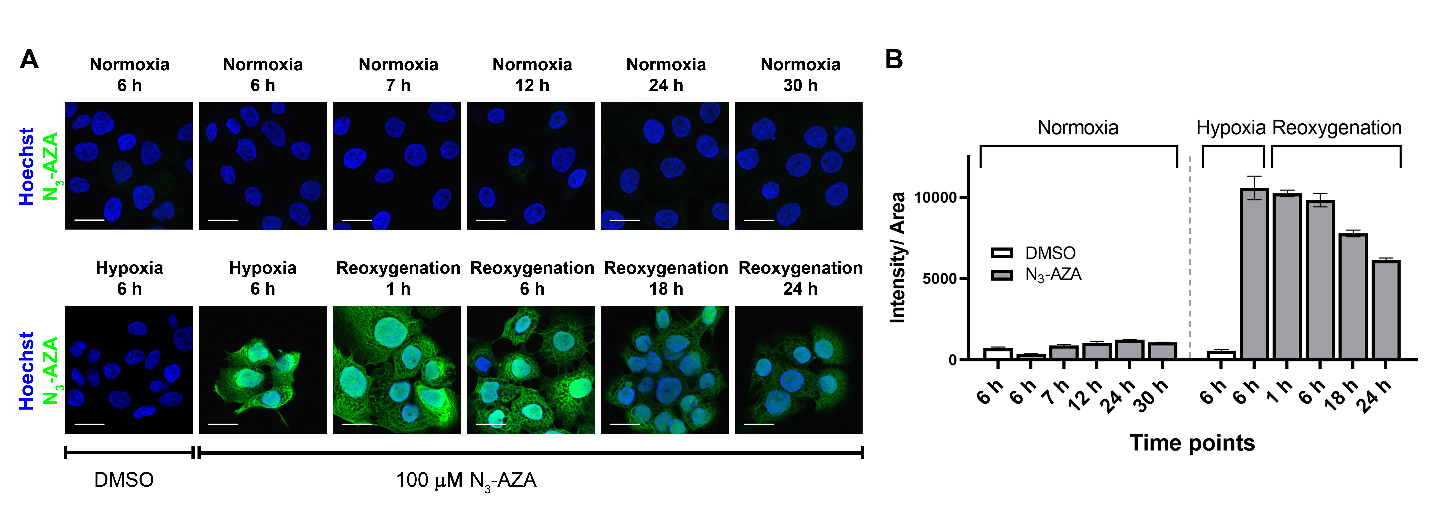
**

**Figure S7.** **N_3_-AZA click staining following reoxygenation.** FaDu cells treated with 100 µM N_3_-AZA (or 0.02% DMSO) were fixed with 2% paraformaldehyde at indicated time points. For hypoxia/ reoxygenation group, cells were fixed either after 6 h of hypoxic incubation or after 1, 6, 18 and 24 h of reoxygenation. Drug was present in the media throughout the reoxygenation period. Fixed cells, processed for N_3_-AZA click chemistry and Hoechst staining, were imaged and quantified as described. N_3_-AZA click staining can be detected in hypoxic reoxygenated cells even after 24 h of reoxygenation (A), however the intensity of the click signal intensity decreases over time (B). Micrographs are representative of at least two independent experiments; scale bar = 20 µm. Quantification shows mean± S.E.M. from three independent replicates.


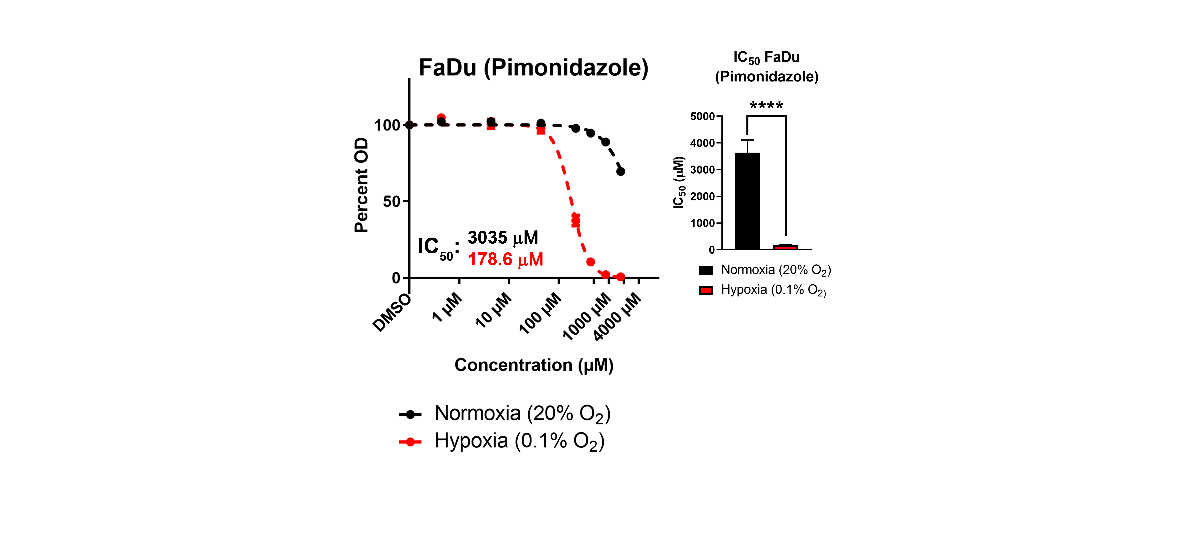


**Figure S8**. **Pimonidazole shows hypoxia selective cytotoxicity.** FaDu cells were treated with increasing concentrations of pimonidazole, incubated for 72 h under normoxia (20% O_2_) and hypoxia (0.1% O­_2_), and crystal violet staining assay was performed. Pimonidazole shows preferential cytotoxicity in hypoxic FaDu cells, with statistically significant differences between their normoxic and hypoxic IC_50_ values. Data represents mean± S.E.M. from at least three independent experiments.


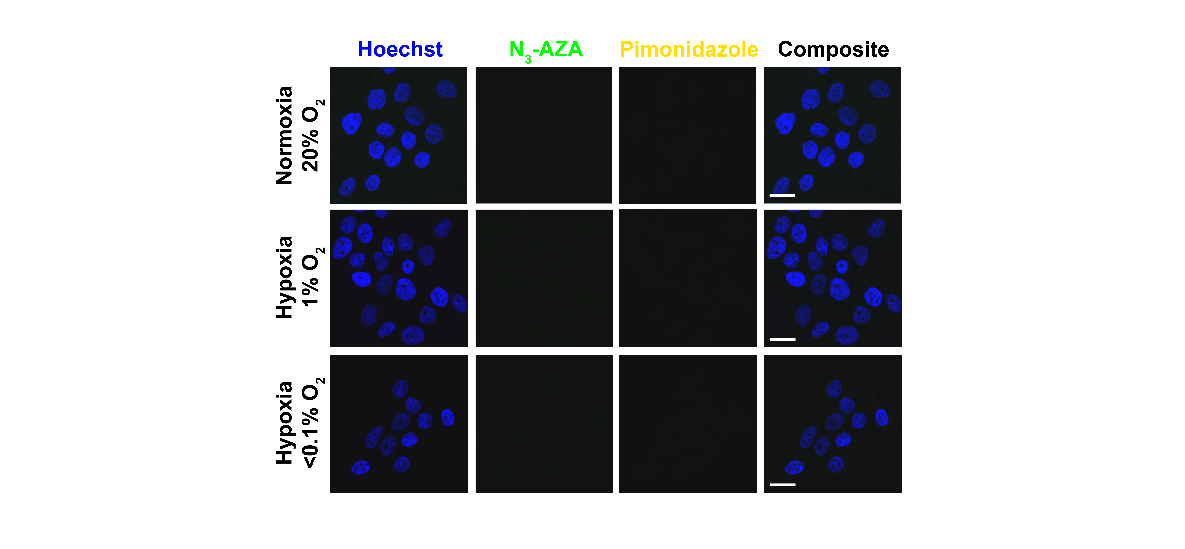


**Figure S9**. **Autofluorescence in N_3_-AZA and pimonidazole channel is minimal.** FaDu cells treated with vehicle control (0.02% DMSO i.e. 0 µM N_3_-AZA and 0 µM pimonidazole) were stained for N_3_-AZA click chemistry and pimonidazole immunostaining. Images were obtained in parallel with cells treated and displayed in Fig. 5A. Minimal fluorescence from N_3­_-AZA and pimonidazole channels was seen in vehicle treated FaDu cells. Representative micrographs are shown from three independent experiments; scale bar = 20 µm.


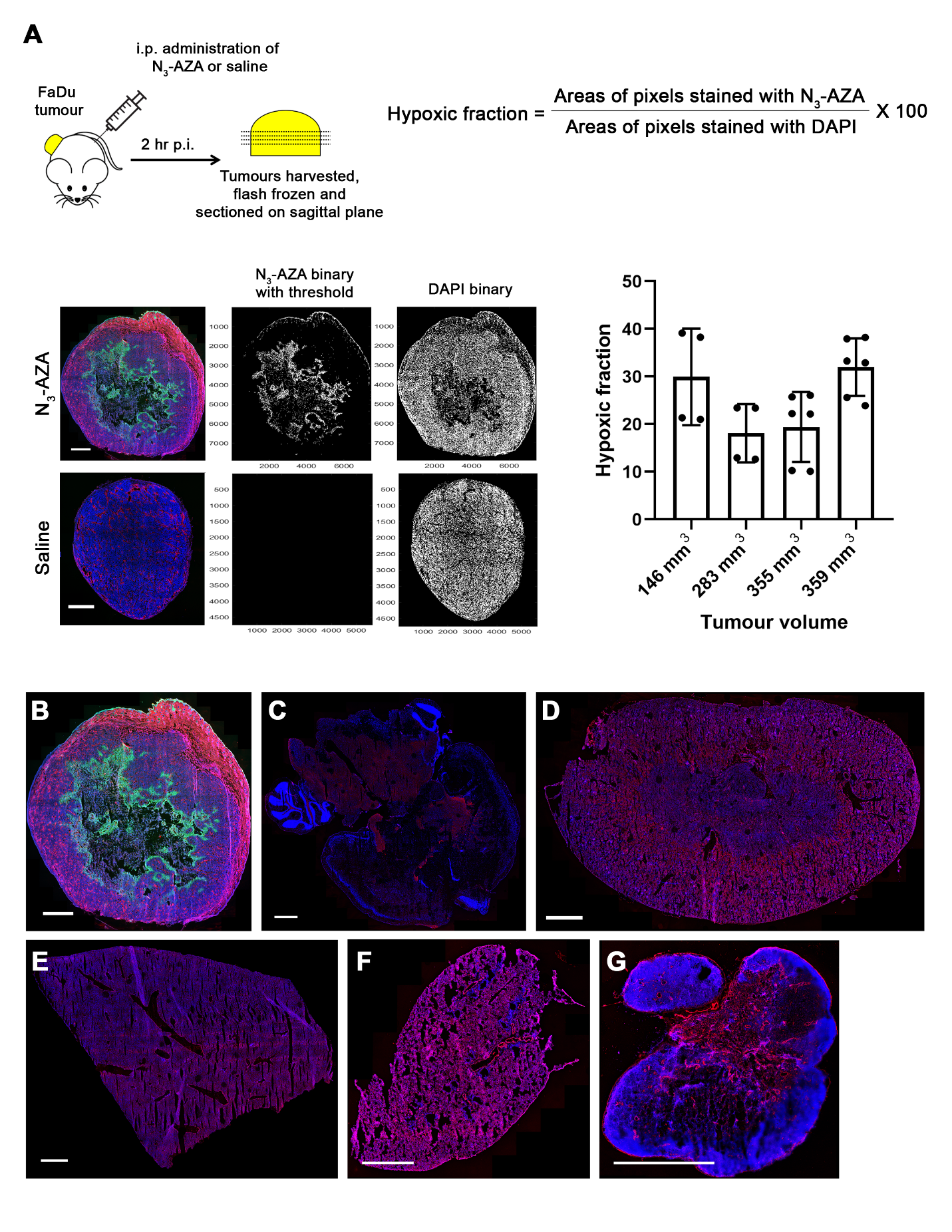


**Figure S10**. **N_3_-AZA click staining is preferentially detected in tumours and can be quantified to determine hypoxic fractions in tumour.** Mice containing subcutaneous FaDu tumors were injected i.p. with N_3_-AZA (or saline) and sacrificed 2 h after injection. Tumours and organs were harvested, and frozen sections were stained for N_3_-AZA click chemistry (hypoxia, green), blood vessels (CD31, red) and nuclei (DAPI, blue). (A) Images were converted to binary using MATLAB; a manual threshold was applied for the click channel and Otsu threshold for DAPI. Hypoxic fraction was quantified using the formula shown. The bar diagram depicts hypoxic fraction across different tumour sizes (means± S.E.M.). At least 4 sections per tumour were stained and quantified independently by two different individuals. N_3_-AZA click staining was detected only in the tumour section (B) whereas different organs [brain (C), kidney (D), liver (E), lung (F) and cervical lymph node (G)] stained negative for N_3_-AZA click chemistry. This points to a preferential uptake of the drug by hypoxic tumour niches. Representative images are shown; scale bar = 1 mm.

**Movie 1**. **N_3_-AZA click staining is concentrated in nucleoli**. FaDu cells, treated with 100 µM N_3_-AZA for 6 h under hypoxia (<0.1% O_2_), were fixed and processed for nucleolin immunostaining (red) and N_3_-AZA click chemistry (green); nuclei were counterstained with Hoechst (blue). Images were obtained in z-stacks with a Plan-Apochromat 40X/1.3 Oil DIC lens on a Zeiss 710 confocal microscope using Zen 2011 software and processed with IMARIS software to prepare a 3D movie. N_3_-AZA click staining was higher in the nucleus, with the signal concentrated in nuclei.

**Movie 2**. **Pimonidazole immunostaining is excluded from nucleoli.** FaDu cells, treated with 100 µM pimonidazole for 6 h under hypoxia (<0.1% O_2_), were fixed and processed for nucleolin immunostaining (red) and pimonidazole immunostaining (yellow); nuclei were counterstained with Hoechst (blue). Images were obtained in z-stacks with a Plan-Apochromat 40X/1.3 Oil DIC lens on a Zeiss 710 confocal microscope using Zen 2011 software and processed with IMARIS software to prepare a 3D movie. Pimonidazole immunostaining was excluded from nucleoli.
